# Pseudomonas aeruginosa Volatilome Characteristics and Adaptations in Chronic Cystic Fibrosis Lung Infections

**DOI:** 10.1128/mSphere.00843-20

**Published:** 2020-10-07

**Authors:** Trenton J. Davis, Ava V. Karanjia, Charity N. Bhebhe, Sarah B. West, Matthew Richardson, Heather D. Bean

**Affiliations:** a School of Life Sciences, Arizona State University, Tempe, Arizona, USA; b Center for Fundamental and Applied Microbiomics, The Biodesign Institute, Tempe, Arizona, USA; c School for Engineering of Matter, Transport, and Energy, Arizona State University, Tempe, Arizona, USA; d Department of Respiratory Sciences, College of Life Sciences, University of Leicester, Leicester, UK; e NIHR Biomedical Research Centre (Respiratory Theme), Institute for Lung Health, Leicester, UK; University of Wisconsin—Madison

**Keywords:** chronic infection, adaptation, *Pseudomonas aeruginosa*, biomarkers, cystic fibrosis, metabolomics, volatile organic compounds

## Abstract

Pseudomonas aeruginosa chronic lung infections in individuals with cystic fibrosis (CF) significantly reduce quality of life and increase morbidity and mortality. Tracking these infections is critical for monitoring patient health and informing treatments. We are working toward the development of novel breath-based biomarkers to track chronic P. aeruginosa lung infections *in situ*. Using comprehensive two-dimensional gas chromatography coupled with time-of-flight mass spectrometry (GC×GC–TOF-MS), we characterized the *in vitro* volatile metabolomes (“volatilomes”) of 81 P. aeruginosa isolates collected from 17 CF patients over at least a 5-year period of their chronic lung infections. We detected 539 volatiles produced by the P. aeruginosa isolates, 69 of which were core volatiles that were highly conserved. We found that each early infection isolate has a unique volatilome, and as infection progresses, the volatilomes of isolates from the same patient become increasingly dissimilar, to the point that these intrapatient isolates are no more similar to one another than to isolates from other patients. We observed that the size and chemical diversity of P. aeruginosa volatilomes do not change over the course of chronic infections; however, the relative abundances of core hydrocarbons, alcohols, and aldehydes do change and are correlated with changes in phenotypes associated with chronic infections. This study indicates that it may be feasible to track P. aeruginosa chronic lung infections by measuring changes to the infection volatilome and lays the groundwork for exploring the translatability of this approach to direct measurement using patient breath.

**IMPORTANCE** Pseudomonas aeruginosa is a leading cause of chronic lung infections in cystic fibrosis (CF), which are correlated with lung function decline. Significant clinical efforts are therefore aimed at detecting infections and tracking them for phenotypic changes, such as mucoidy and antibiotic resistance. Both the detection and tracking of lung infections rely on sputum cultures, but due to improvements in CF therapies, sputum production is declining, although risks for lung infections persist. Therefore, we are working toward the development of breath-based diagnostics for CF lung infections. In this study, we characterized of the volatile metabolomes of 81 P. aeruginosa clinical isolates collected from 17 CF patients over a duration of at least 5 years of a chronic lung infection. We found that the volatilome of P. aeruginosa adapts over time and is correlated with infection phenotype changes, suggesting that it may be possible to track chronic CF lung infections with a breath test.

## Abstract (precis)

Pseudomonas aeruginosa is a leading cause of chronic lung infections in cystic fibrosis (CF), which are correlated with lung function decline. Significant clinical efforts are therefore aimed at detecting infections and tracking them for phenotypic changes, such as mucoidy and antibiotic resistance. Both the detection and tracking of lung infections rely on sputum cultures, but due to improvements in CF therapies, sputum production is declining, although risks for lung infections persist. Therefore, we are working toward the development of breath-based diagnostics for CF lung infections. In this study, we characterized of the volatile metabolomes of 81 P. aeruginosa clinical isolates collected from 17 CF patients over a duration of at least 5 years of a chronic lung infection. We found that the volatilome of P. aeruginosa adapts over time and is correlated with infection phenotype changes, suggesting that it may be possible to track chronic CF lung infections with a breath test.

## INTRODUCTION

Cystic fibrosis (CF) is an autosomal recessive disease caused by a mutation in the CFTR protein that regulates ion transport across epithelia. In the lungs, reduced or lost CFTR function leads to defective mucociliary transport, facilitating infection and colonization by a plethora of microorganisms (1). Pseudomonas aeruginosa is one of the most prevalent lung pathogens in CF—especially after adolescence (2)—and is able to establish chronic infections that can last for years to decades (3, 4). P. aeruginosa lung infection is associated with more rapid lung function decline, increased risk of hospitalization, and increased risk of death (5, 6). Significant clinical efforts are therefore aimed at diagnosing and treating new infections to delay the onset of chronic infection (7).

As P. aeruginosa transitions from an acute infection to chronicity, it undergoes a variety of genotypic, phenotypic, and metabolic changes (8, 9). It has been well established that several phenotypes are correlated with chronic infection, including reduced motility and increased mucoidy, antibiotic resistance, and biofilm formation (9–11), and some of these phenotypes are also correlated with poorer patient outcomes (6, 12, 13). In light of this, P. aeruginosa phenotypes could be used to evaluate infection stage, CF disease progression, and patient morbidity risk (10, 12, 14, 15). Poor access to infection sites in the lower airways, however, delays the detection of new P. aeruginosa infections and reduces the feasibility and accuracy of disease state tracking via bacterial phenotypes, whether by culture-dependent or culture-independent methods (16).

We are developing breath-based diagnostics to detect new P. aeruginosa infections and track chronic infection phenotypes in the CF lung. Breath contains thousands of volatile organic compounds capable of conveying a wealth of information about human health and disease (17, 18), and it is being leveraged for the development of diagnostics for wide-ranging conditions (19, 20). In the context of chronic lung infections, breath-based diagnostics possess significant advantages over other diagnostic modalities. First, breath provides a noninvasive way of sampling the entire ventilated lung, a limitation of both sputum and bronchoalveolar lavage fluid that leads to delays in diagnosis of new infections and detection of infection phenotype changes (16, 21–24). Second, breath sampling captures metabolic information about the pathogens *in situ*, eliminating the need for microbial enrichment steps and speeding the time to diagnosis by days to weeks (25, 26). Additionally, *in situ* metabolic measurements provide information on the physiology of the pathogens in the context of disease versus the context of the lab. We propose that a breath-based diagnostic can be created for detecting and tracking CF P. aeruginosa lung infections by correlating the microbial genotypic and phenotypic changes that occur during chronic infections to changes in the infection volatile metabolome, or “volatilome.”

In order to develop a set of biomarkers for diagnosing and tracking P. aeruginosa lung infections in CF, we must consider the genomic, phenomic, and volatilomic diversity of the species and how that diversity may be altered via adaptation to the CF lung environment. A recent effort to sequence more than 1,300 P. aeruginosa isolates demonstrates that the genomes are highly flexible, with only 1% of the pan-genome being conserved across all isolates (27). An untargeted analysis of the volatile metabolites produced *in vitro* by 24 P. aeruginosa clinical isolates shows that the volatilomes are also diverse, with 18% of the pan-volatilome being conserved in this comparatively small study (28). In the context of CF, most early P. aeruginosa infections in young patients are caused by unique strains that come from the patients’ environments (29–32). We therefore expect the volatilomes early in infection to have high interpatient dissimilarity, reflecting the genomic diversity of P. aeruginosa CF isolates (3, 33–35), and to be chemically diverse (i.e., volatiles from a wide range of chemical classes). Once chronic infections are established, however, the “founder” P. aeruginosa strain evolves into a population of clonal substrains (3, 8, 36–40), many of which harbor CF-typical loss-of-function mutations in regulatory genes and sigma factors (e.g., *lasR* and *mucA*) (3, 41, 42). From this, we posit that the size of the P. aeruginosa volatilome will shrink during chronic infection, with a concomitant decrease in chemical diversity and a reduction in interpatient dissimilarity.

The primary goal of this study was to build a foundation for using volatilomes to diagnose and track P. aeruginosa CF lung infections by exploring how the *in vitro* volatilome of P. aeruginosa CF isolates changes over the course of chronic infection. We used comprehensive two-dimensional gas chromatography coupled with time-of-flight mass spectrometry (GC×GC–TOF-MS) to characterize the volatilomes of 81 P. aeruginosa isolates from early and late chronic lung infections from 17 persons with CF. Using an untargeted metabolomics approach, we characterized the size and chemical composition of the CF P. aeruginosa volatilome and identified core volatiles that would be a primary source of breath biomarkers for diagnosing infections. We also investigated how the P. aeruginosa volatilome is shaped by the CF lung environment, characterizing changes in volatilome sizes and compositions over time, and how these changes relate to intrapatient and interpatient volatilome dissimilarities. A secondary goal of this study was to provide further data on the P. aeruginosa volatilome via the largest single analysis of P. aeruginosa headspace volatiles to date.

## RESULTS

### Characteristics of P. aeruginosa CF isolates.

We obtained from a biorepository 81 P. aeruginosa chronic infection isolates, which had been collected from 17 individuals with CF. From 14 patients, two or three P. aeruginosa isolates were obtained: one isolate that was the first cultured P. aeruginosa strain and one or two isolates that were collected at least 5 years after the first. We refer to these isolates as early and late isolates, respectively. For one patient (patient 75), four isolates were collected: two early isolates collected 1 month apart and two late isolates collected 10 and 16 years after the first. For one patient (patient 23), 32 additional isolates were collected over the course of a 7.5-year infection period, which we termed intermediate isolates. For one patient, only one isolate was collected. These isolates were genetically characterized in a study by Smith et al., who determined that the intrapatient replicate isolates are all clonally related, with the exception of the four isolates from patient 75, which are actually two clonally related early/late pairs (isolates 75E-1 and 75L-2 are clonal, and isolates 75E-2 and 75L-1 are clonal) (3).

**FIG 1:**
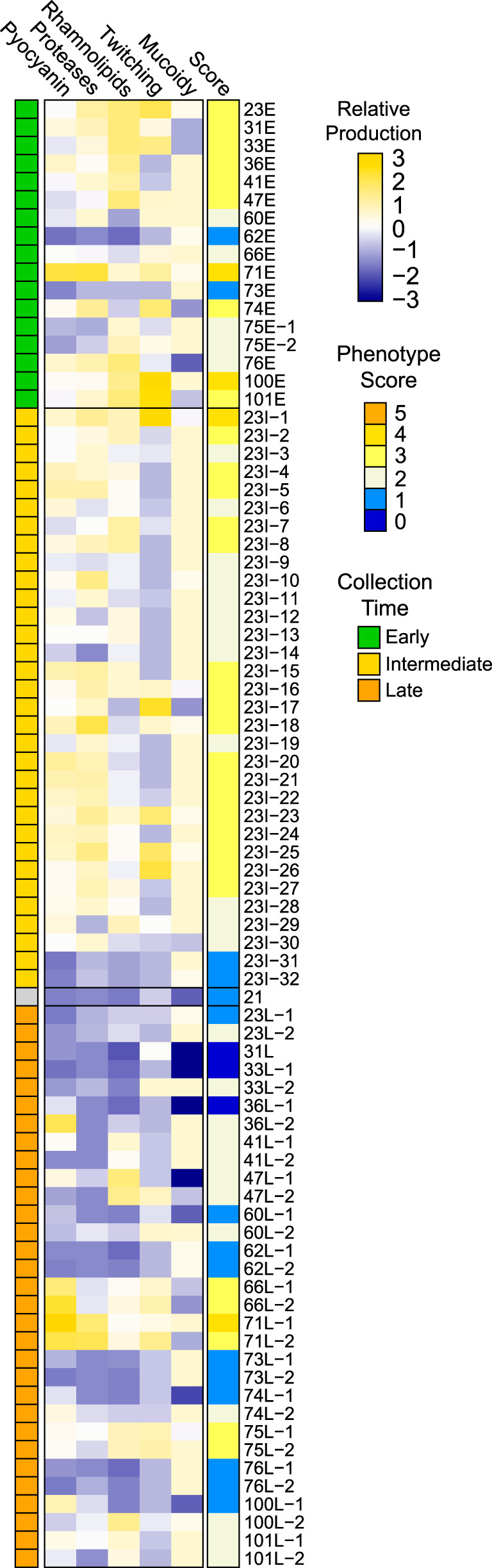
Relative phenotype production of 81 P. aeruginosa CF chronic lung infection isolates. Each phenotype was independently scaled to a range of 0 to 1, with 0 representing the minimum data point and 1 representing the maximum data point (excluding outliers), and the relative productions are depicted in the heat map as standardized values (mean-centered and scaled to unit variance) across isolates. Phenotype score is the sum of the scaled, nonstandardized phenotype values, rounded to the nearest integer.

For all isolates, we characterized five clinically relevant phenotypes *in vitro*: mucoidy, pyocyanin production, rhamnolipid production, protease production, and twitching motility (Fig. 1; Table S1). These phenotypes are commonly altered during the course of chronic CF lung infections (15). There were wide ranges in the expression of these phenotypes across all isolates, as expected. Several isolate sets exhibited phenotypes consistent with age of collection, in that early isolates possessed higher degrees of motility, higher quantities of pyocyanin, rhamnolipids, and proteases (indicating intact quorum regulation) and lower mucoidy compared to their cognate late isolates (e.g., those from patients 23, 31, 33, 36, and 76). The associations between relative patient age at isolate collection and P. aeruginosa phenotypes were not perfect, however. For example, early isolate 62E had no detectable pyocyanin, proteases, rhamnolipids, or twitching motility, and many of the late isolates retained more early-like phenotypes (e.g., both late isolates from patients 41, 66, 71, 75, and 101, and 60L-1, 74L-2, and 100L-2). Despite observable changes in isolate phenotype within a patient, no phenotypes were significantly different when the late isolate(s) was compared to the early isolate. Collectively, the 81 isolates were highly varied and represented the array of phenotypes we expect to observe in the span of P. aeruginosa CF lung infections from initial infection to long-established chronic infections.

10.1128/mSphere.00843-20.8 TABLE S1 Summary statistics of 81 P. aeruginosa isolates collected from 17 patients with CF. Isolate 21 was the only isolate from that patient included in this study. E, isolate collected early in chronic infection; L, isolate collected late in chronic infection; I, isolate collected intermediately between the early and late isolates. AGE, age in years of patient when isolate was collected. PYO, pyocyanin (absorbance); PRO, protease (cm); RHL, rhamnolipids (score); TWI, twitching motility (cm); MUC, mucoidy (visual score). See Materials and Methods for information on the assays used to collect phenotype data, which are presented in the table as raw, nonscaled values that are the means for three biological replicates. Score was determined for each replicate by scaling the data for each phenotype to a range of 0 to 1, where 1 is the maximum value and 0 is the minimum value (excluding outliers). Scaled phenotype data were then summed to yield the score. Age is not included in calculation of scores. The score reported in the table is the mean of triplicate scores for three biological replicates, rounded to the nearest integer. *P* values indicate the significance of the late-isolate score compared to the early-isolate score using the Wilcoxon signed-rank test (with continuity correction where appropriate). Isolates selected for further early versus late analyses are indicated with asterisks. Download Table S1, PDF file, 0.1 MB. Copyright © 2020 Davis et al. 2020 Davis et al. This content is distributed under the terms of the Creative Commons Attribution 4.0 International license.

### *In vitro* volatilome of P. aeruginosa CF isolates.

Taking an untargeted metabolomics approach, we cultured the clinical P. aeruginosa isolates *in vitro* and characterized their volatilomes using GC×GC–TOF-MS. Following extensive data processing, including removal of analytically biased chromatographic features, we conservatively attributed 539 nonredundant volatile compounds to the growth and metabolism of the P. aeruginosa isolates (Table S2). Among these, 69 compounds are shared by at least 95% (*n* ≥ 77) of the isolates, representing core volatiles. Using minimum metabolomics reporting standards, we assigned compound identification levels 1 to 4 (with 1 being high; 2 was the highest level in this study) based on a combination of mass spectral and chromatographic characteristics. We were able to assign putative names to 14 core and 33 noncore compounds with an ID level of 2 (Table 1), many of which have been previously reported as P. aeruginosa volatiles (Table 1). While we detected 2-aminoacetophenone (the volatile compound responsible for the grape-like odor of P. aeruginosa), it possessed an intraclass correlation slightly lower than the applied threshold of 0.75 and was ultimately filtered out of our peak list. We also identified several compounds from a variety of chemical classes that have not been previously reported for P. aeruginosa, including alcohols (3-methyl-1-buten-1-ol, 1-butoxy-2-propanol, and 2-butyl-1-octanol), esters (butanoic acids and methyl isovalerate), aromatics [tetrahydrofuran, tetramethylpyrazine, 2-butylfuran, 3-cyano-2,5-dimethylpyrazine, and 2,4(1,1-dimethyl)-phenol], unsaturated ketones [3-hydroxy-butan-2-one, 4-undecanone, 1-octen-3-one, 3-penten-2-one, 6-methyl-2-heptanone, and 1-(4-ethylphenyl)-ethanone], and an aldehyde (decanal).

**TABLE 1:** Putatively identified volatile compounds metabolized by P. aeruginosa CF isolates

Compound name^*a*^	Formula	Chemical class	No. of samples (*n* = 81)	Reference(s)^*b*^
Core volatile compounds				
**3-Methylbutanal**	C_5_H_10_O	Aldehyde	79	
Thiocyanic acid, methyl ester	C_2_H_3_NS	Ester	77	43
3-Methyl-1-butanol	C_5_H_12_O	Alcohol	77	44, 46, 47
**Pyridine**	C_5_H_5_N	Aromatic	77	
**3-Penten-2-one**	C_5_H_8_O	Ketone	77	
**3-Methyl-1-buten-1-ol**	C_5_H_10_O	Alcohol	78	
4-Methyl-3-penten-2-one	C_6_H_10_O	Ketone	81	28, 48
Hexanal	C_6_H_12_O	Aldehyde	81	44, 46, 48
**Heptanal**	C_7_H_14_O	Aldehyde	80	
2,5-Dimethylpyrazine	C_6_H_8_N_2_	Aromatic	81	43
2-Nonanone	C_9_H_18_O	Ketone	81	28, 43–45, 47–53
**4-Ethyl-1,2-dimethylbenzene**	C_10_H_14_	Aromatic	77	
**1,3,3-Trimethyl-(bicyclo)-heptan-2-ol**	C_10_H_18_O	Alcohol	78	
**1-(4-Ethylphenyl)-ethanone**	C_10_H_12_O	Ketone	79	

Noncore volatile compounds				
Dimethyl sulfide	C_2_H_6_S	Other	29	28, 43, 44, 46, 47, 50, 54, 55
**2-Methyl-3-buten-2-ol**	C_5_H_10_O	Alcohol	35	
2-Butanone	C_4_H_8_O	Ketone	1	28, 43–45, 47, 49, 52, 56
**Tetrahydrofuran**	C_4_H_8_O	Aromatic	1	
**Acetic acid**	C_2_H_4_O_2_	Carboxylic acid	55	
Methyl thioacetate	C_3_H_6_OS	Thiol	3	44, 46, 48
2,4-Dimethylfuran	C_6_H_8_O	Aromatic	66	28
**Butanoic acid, methyl ester**	C_5_H_10_O_2_	Ester	3	
**3-Hydroxybutan-2-one**	C_4_H_8_O_2_	Ketone	18	
Dimethyl disulfide	C_2_H_2_S_2_	Other	1	28, 44–48, 52, 54–59
**2-Methyl-butanoic acid, methyl ester**	C_6_H_12_O_2_	Ester	3	
**3-Methyl-butanoic acid, methyl ester**	C_6_H_12_O_2_	Ester	3	
Furfural	C_5_H_4_O_2_	Aromatic	67	46
**2-Butylfuran**	C_8_H_12_O	Aromatic	6	
2-Hexen-1-ol	C_6_H_12_O	Alcohol	51	28
**1-Butoxy-2-propanol**	C_7_H_16_O_2_	Alcohol	61	
**6-Methyl-2-heptanone**	C_8_H_16_O	Ketone	33	
**1-Methylethylbenzene**	C_9_H_12_	Aromatic	57	
3-Octanone	C_8_H_16_O	Ketone	56	28, 47
**4,6-Dimethyl-2-heptanone**	C_9_H_18_O	Ketone	47	
**2-Ethyl-5-methylpyrazine**	C_7_H_10_N_2_	Aromatic	58	
4-Nonanone	C_9_H_18_O	Ketone	49	28
**Tetramethylpyrazine**	C_8_H_12_N_2_	Aromatic	5	
2-Octenal	C_8_H_14_O	Aldehyde	48	43
Octanenitrile	C_8_H_15_N	Other	73	28
2,4-Octadienal	C_8_H_12_O	Aldehyde	9	28
3-Decanone	C_10_H_20_O	Ketone	41	43
**Decanal**	C_10_H_20_O	Aldehyde	65	
2-Decanone	C_10_H_20_O	Ketone	13	28
**4-Undecanone**	C_11_H_22_O	Ketone	30	
2-Dodecanone	C_12_H_24_O	Ketone	18	28, 43
**2-Butyl-1-octanol**	C_12_H_26_O	Alcohol	48	
**2,4-bis(1,1-Dimethyl)-phenol**	C_14_H_22_O	Aromatic	26	

a Compounds in bold type have not been previously reported as P. aeruginosa volatile compounds.

b References reporting these volatiles in P. aeruginosa cultures.

10.1128/mSphere.00843-20.9 TABLE S2 Table of 539 peaks metabolized by 81 P. aeruginosa clinical CF isolates. Download Table S2, XLSX file, 0.05 MB. Copyright © 2020 Davis et al. 2020 Davis et al. This content is distributed under the terms of the Creative Commons Attribution 4.0 International license.

**FIG 2:**
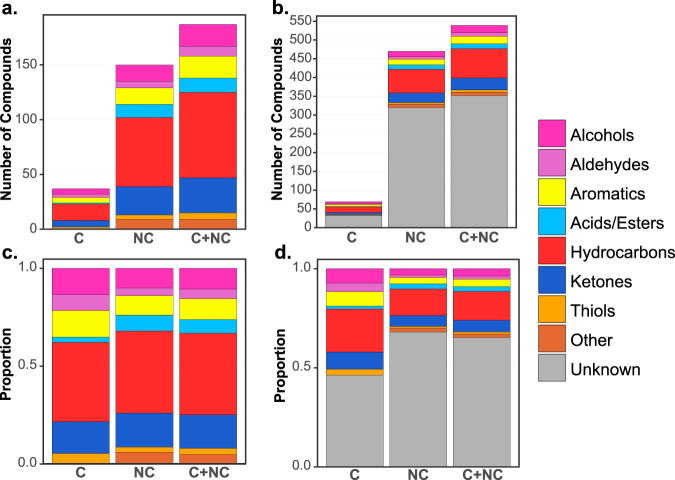
Size and chemical composition of volatile compounds metabolized by 81 P. aeruginosa CF chronic infection isolates. (a) Identified compounds; (b) identified and unknown compounds; (c) identified compounds, scaled to 100%; (d) identified and unknown compounds, scaled to 100%. C, core compounds, defined as those detected in 95% or more of samples; NC, noncore compounds; C+NC, sum of core and noncore compounds.

Chemical classifications were assigned to ID level 2 and 3 compounds (187 compounds) using mass spectral match and retention time characteristics (Fig. 2). Among level 2 and 3 core volatiles, the majority of classified compounds were hydrocarbons (41%), followed by ketones (16%). Other oxidized compounds, including alcohols, aromatics, acids, and thiols, accounted for an additional 43%. The noncore volatiles had similar chemical compositions, which are consistent with previous studies of the P. aeruginosa volatilome (25, 28, 43–45). Of the entire volatilome, 65% of the volatiles (*n *= 352) possessed less than an 80% mass spectral match to the 2011 NIST MS library and as such were classified as unknowns.

### Relationships in the P. aeruginosa CF volatilome within and between patients.

**FIG 3:**
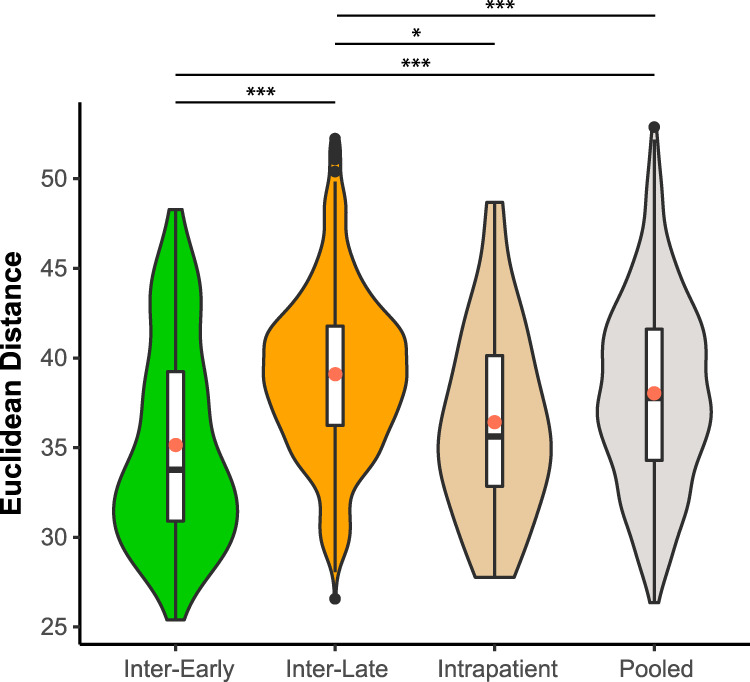
Violin plots and box plots of pairwise dissimilarities (defined as Euclidean distance) of P. aeruginosa chronic infection isolate volatilomes. “Inter-Early” indicates between-patient early-early isolate dissimilarities. “Inter-Late” indicates between-patient late-late isolate dissimilarities. “Intra-Patient” indicates within-patient early-late isolate dissimilarities. “Pooled” represents all pairwise volatilome dissimilarities. Red circles indicate the means. Significant differences between means were identified using one-way ANOVA with Tukey’s HSD test. *, *P* < 0.05; ***, *P* < 0.001.

Because every patient is infected by a genetically unique strain, we hypothesized that the dissimilarities of intrapatient volatilomes would be lower than those of interpatient volatilomes. Additionally, due to the greater metabolic potential of early-infection isolates with intact regulatory networks, we hypothesized that the volatilomes of interpatient early isolates would be more dissimilar than those of interpatient late isolates. We calculated the pairwise Euclidean distances as a measure of dissimilarity between all early isolate volatilomes (“inter-early dissimilarity”), all late isolate volatilomes (“inter-late dissimilarity”), and between early and late isolate volatilomes from the same patient (“intrapatient dissimilarity”). Significant differences between means were tested using one-way analysis of variance (ANOVA) with Tukey’s honestly significant differences (HSD) multiple comparison procedure. Interestingly, the inter-early dissimilarity was the lowest (mean, 35.2; median, 33.8), and the inter-late dissimilarity was the highest (mean, 39.1; median, 39.1), and these differences were significant (95% confidence interval [CI], 3.05 to 4.86; *P = *0+) (Fig. 3). Similarly, mean inter-late volatilome dissimilarity was significantly greater than that of intra-patient volatilomes (mean, 36.4; median, 35.6) (95% CI, 0.41 to 4.97; *P = *0.013). Even more intriguing was that the inter-early dissimilarity was lower than even the intrapatient dissimilarity, though this difference was not significant (95% CI, −1.11 to 3.66; *P = *0.515). For comparison, we also calculated the pairwise Euclidean distances of all isolates in this study (pooled; mean, 38.0; median, 37.9); the mean inter-early and mean inter-late dissimilarities were both significantly lower than the mean dissimilarity of the pooled isolates (95% CIs, 1.99 to 3.77 and −1.65 to 0.51; *P = *0+).

**FIG 4:**
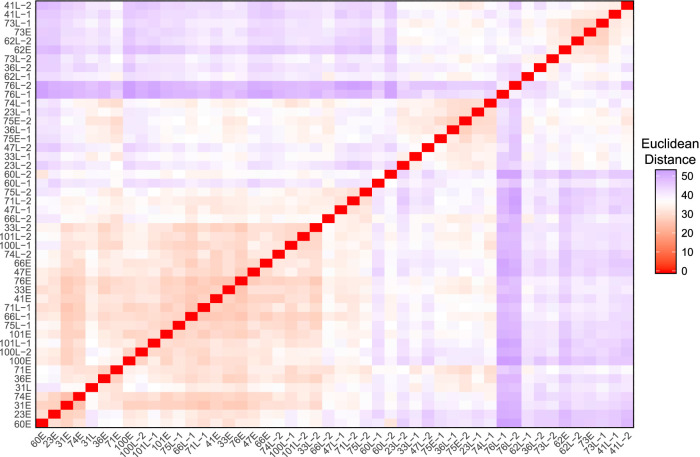
Ordered dissimilarity image (ODI) of the truncated set of 48 P. aeruginosa clinical isolates depicting volatilome dissimilarity defined by Euclidean distance.

Visualizing the Euclidean distances between isolate volatilomes via nonmetric multidimensional scaling (NMDS) and ordered dissimilarity images (ODI), we looked for clustering of clonal isolates, which indicates intrapatient similarities. The NMDS and ODI of the Euclidean distances of all 81 isolate volatilomes show a primary cluster composed mostly of intermediate isolates of patient 23 (Fig. S1). To reduce the influence of the overrepresented patient 23 isolates in data visualization, we truncated our data set to 48 isolates by removing the intermediate isolates of patient 23 and the lone patient 21 isolate. The ODI plot and NMDS of the truncated set revealed three clusters of similarity, none of which include all isolates from a single patient (Fig. 4; Fig. S2a and b). This refutes our hypothesis that isolates from the same patient maintain a similar volatilome over time. To determine if any interpatient or intrapatient isolate pairs are significantly different, we performed permutational multivariate analysis of variance (PERMANOVA) on the Euclidean distances for all possible pairs. Following Benjamini-Hochberg adjustment of *P* values, no pairs of isolates were identified as having significantly different volatilomes (*q *≥ 0.1 for all isolate pairs). However, PERMANOVA conducted on all isolates indicated strong significant differences between the 81 volatilomes (pseudo-*F*_80,161_ = 28.0; *P < *0.01), as well as between the volatilomes of the truncated set of 48 isolates (pseudo-*F*_47,96_ = 29.2; *P = *0+), reinforcing the inference from the ODI and NMDS that the CF P. aeruginosa volatilome is heterogeneous.

10.1128/mSphere.00843-20.1 FIG S1 Nonmetric multidimensional scaling (NMDS) plots of 81 P. aeruginosa clinical isolates depicting volatilome dissimilarity defined by Euclidean distance colored by collection time (a) and colored by patient (b). (c) Ordered dissimilarity image (ODI) of 81 P. aeruginosa clinical isolates depicting volatilome dissimilarity defined by Euclidean distance. Download FIG S1, JPG file, 0.7 MB. Copyright © 2020 Davis et al. 2020 Davis et al. This content is distributed under the terms of the Creative Commons Attribution 4.0 International license.

10.1128/mSphere.00843-20.2 FIG S2 Nonmetric multidimensional scaling (NMDS) plots of the truncated set of 48 P. aeruginosa clinical isolates depicting volatilome dissimilarity defined by Euclidean distance colored by collection time (a) and colored by patient (b). (c) NMDS plot of the 35 P. aeruginosa clinical isolate volatilomes from patient 23 depicting volatilome dissimilarity defined by Euclidean distance. Download FIG S2, JPG file, 0.3 MB. Copyright © 2020 Davis et al. 2020 Davis et al. This content is distributed under the terms of the Creative Commons Attribution 4.0 International license.

**FIG 5:**
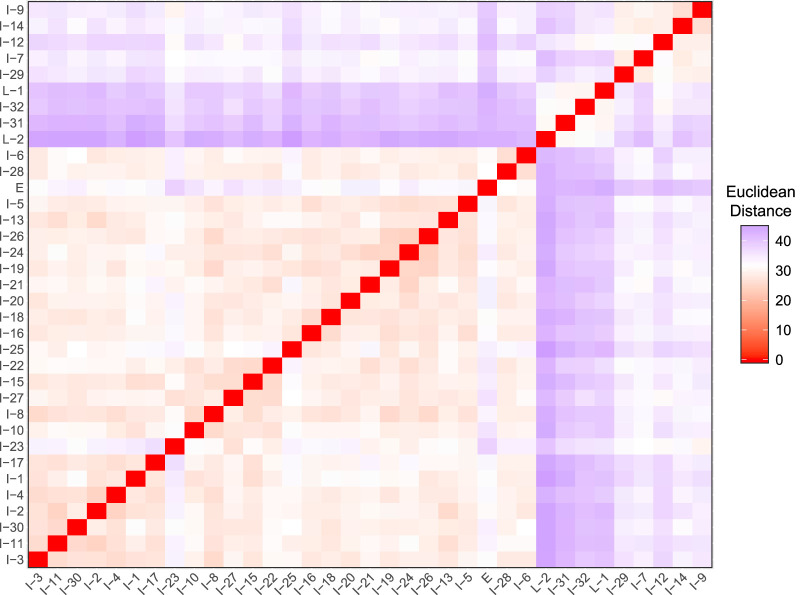
Ordered dissimilarity image (ODI) of the 35 P. aeruginosa clinical isolates from patient 23 depicting volatilome dissimilarity defined by Euclidean distance. For visual clarity, the isolate labels do not include the patient number.

**FIG 6:**
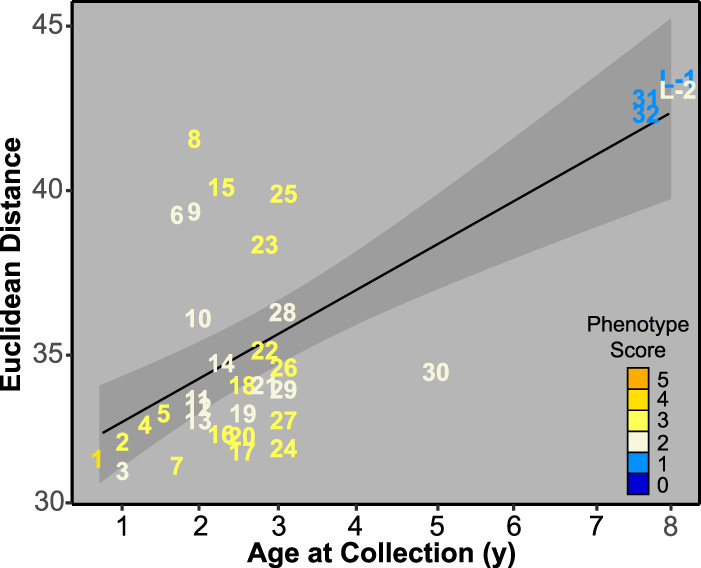
Dissimilarity of patient 23 volatilomes over time, defined as Euclidean distance from the early isolate (not shown). For visual clarity, the isolate labels do not include the patient number and intermediate isolate labels have been truncated to include only the numeral that signifies collection order (e.g., I-1 is represented as 1). The black line represents the linear regression fit (*r* = 0.70; *P = *0+), and the shaded region represents the standard error of the regression line.

Though we did not find that patient isolates maintain similar volatilomes over the duration of chronic infection, it is noteworthy that the majority of patient 23’s isolates have strong similarities (Fig. S1). Taking a closer look at the 35 isolates from patient 23 (Fig. 5; Fig. S2c), the ODI and NMDS revealed four distinct similarity neighborhoods: one neighborhood consisting of only the early isolate, 23E, which stands alone; a second, consisting of the four latest-collected isolates, 23I-32, 23I-33, 23L-1, and 23L-2; a third, consisting of isolates 23I-7, 23I-9, 23I-12, 23I-14, and 23I-29; and a fourth, encompassing the remainder of the isolates. The four latest-collected isolates are less defined by their similarity to each other than their dissimilarity to all other isolates collected during the infection. A linear regression of the Euclidean distances between the early isolate and the intermediate and late isolates as a function of patient age showed that isolates became increasingly dissimilar to the early isolate over time (Pearson’s *r* = 0.70; *P = *0+) (Fig. 6). These observations are underpinned by the phenotypic differences of the four latest isolates and the number and types of known mutations that were accumulated in the 23L versus 23E isolates (3). As described by Smith et al. (3), the P. aeruginosa infection sampled from patient 23 (referred to as patient 1 in the referenced publication) diversified from a patient age of 1.5 to 3 years into a population of isolates, with mutations in virulence, motility, quorum sensing, iron transport, efflux, and transcription and translation genes. The volatilome dissimilarities we measured reflect the genotypic diversification of the infection as a function of time (Fig. 6).

### Chemical characteristics of the volatilomes of early and late isolates.

**FIG 7:**
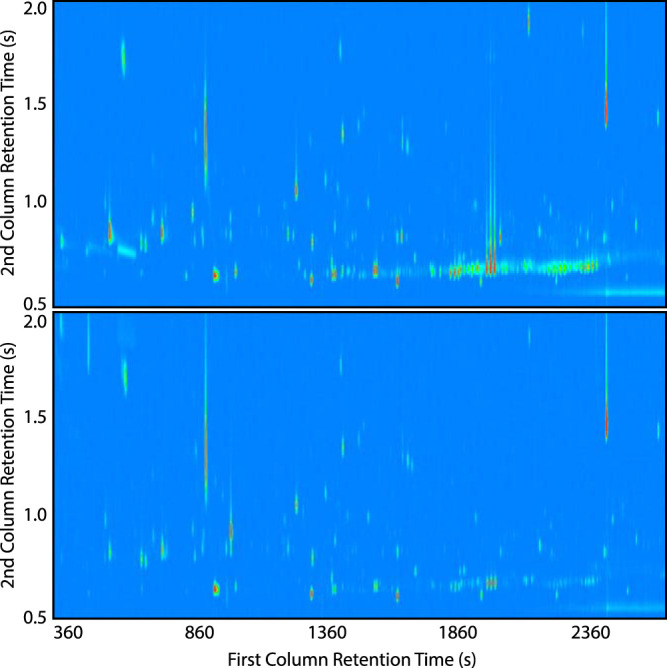
Representative GC×GC chromatograms of early (top) and late (bottom) chronic infection isolates (41E and 41L-2 are depicted). Dark blue represents the baseline, and peak intensity is depicted using a color gradient from light blue (low) to dark red (high). Chromatographic regions where ^1^*t*_R_ is <200 s and ^2^*t*_R_ is <0.5 s were excluded for visual clarity. All chromatograms for the 10 selected early and late isolate pairs are provided in Miscellaneous Information at https://doi.org/10.6084/m9.figshare.12990908.

We compared the chemical characteristics of the early and late volatilomes, positing that the size and chemical diversity of the volatilomes would decrease from early to late infection due to loss-of-function mutations. To perform the comparison, we needed to balance the size of the isolate groups, and we did so by selecting 10 pairs of P. aeruginosa isolates that had large changes in phenotypes over the duration of infection, thereby enhancing the differences we might find in the early versus late volatilomes (Fig. 1; Table S1). Visual inspection of the 10 pairs of GC×GC chromatograms suggests reductions in the number and variety of volatile compounds produced by late isolates (Fig. 7; also, see Fig. 2 in Miscellaneous Information [https://doi.org/10.6084/m9.figshare.12990908]). Contrary to the appearance of the chromatograms, however, the overall size and chemical compositions of the early and late volatilomes were similar to each other (Fig. S3a to d), with hydrocarbons representing approximately 50% and alcohols and ketones together representing approximately 30% of the 410 early and 441 late volatiles. We quantified the chemical richness and diversity of the pooled early and pooled late isolates using the Shannon-Wiener diversity index (Table S3) and found that the volatilomes of early and late isolates were similar, whether all volatiles or only the noncore volatiles were used.

10.1128/mSphere.00843-20.3 FIG S3 Size and chemical composition of volatile compounds metabolized by the selected 10 early-late pairs of P. aeruginosa chronic infection CF isolates (a-d). (a) Identified compounds; (b) identified and unknown compounds; (c) identified compounds, scaled to 100%; (d) identified and unknown compounds, scaled to 100%. Size and chemical composition of volatile compounds metabolized by each of the 10 early-late pairs of P. aeruginosa chronic infection CF isolates (e-h). (e) Identified compounds; (f) identified and unknown compounds; (g) identified compounds, scaled to 100%; (h) identified and unknown compounds, scaled to 100%. C, core compounds, defined as those detected in 95% or more of samples; NC, noncore compounds; C+NC, sum of core and noncore compounds. Download FIG S3, JPG file, 0.7 MB. Copyright © 2020 Davis et al. 2020 Davis et al. This content is distributed under the terms of the Creative Commons Attribution 4.0 International license.

10.1128/mSphere.00843-20.10 TABLE S3 Measures of richness and Shannon diversity of the selected 10 early-late pairs of P. aeruginosa clinical CF isolate volatilomes. Download Table S3, PDF file, 0.09 MB. Copyright © 2020 Davis et al. 2020 Davis et al. This content is distributed under the terms of the Creative Commons Attribution 4.0 International license.

The uniformity in the early versus late volatilomes is not a function of data aggregation, as the volatilomes of the individual isolates are highly similar in number and chemical composition, with the exception of one late isolate (76L) that has a slow-growth phenotype (Fig. S3e to h). Furthermore, when individual isolate pairs were examined, for the vast majority there were no differences in the richness and diversity of their volatilomes (Table S3). Together, these results suggest that there are not major differences between the number and diversity of chemical compounds between early and late isolates. Hierarchical clustering analysis (HCA) of the P. aeruginosa isolates by their volatilomes underscores this observation; we observed no discernible clustering of the early versus late isolates based on the presence and absence of volatiles (Fig. S4a).

10.1128/mSphere.00843-20.4 FIG S4 Hierarchical clustering analysis (HCA) of the 10 early-late pairs of P. aeruginosa clinical CF isolates, based on (a) presence and absence and (b) relative abundance of 472 volatile compounds. Core compounds are present in at least 95% of isolates; noncore compounds are present in less than 95%. Volatiles are in columns. Clustering is based on rows (isolates), which are color coded by their phenotype score (left color block) and relative time of collection (right color block). Download FIG S4, JPG file, 1.1 MB. Copyright © 2020 Davis et al. 2020 Davis et al. This content is distributed under the terms of the Creative Commons Attribution 4.0 International license.

### Abundance of volatile compounds in early and late isolates.

**FIG 8:**
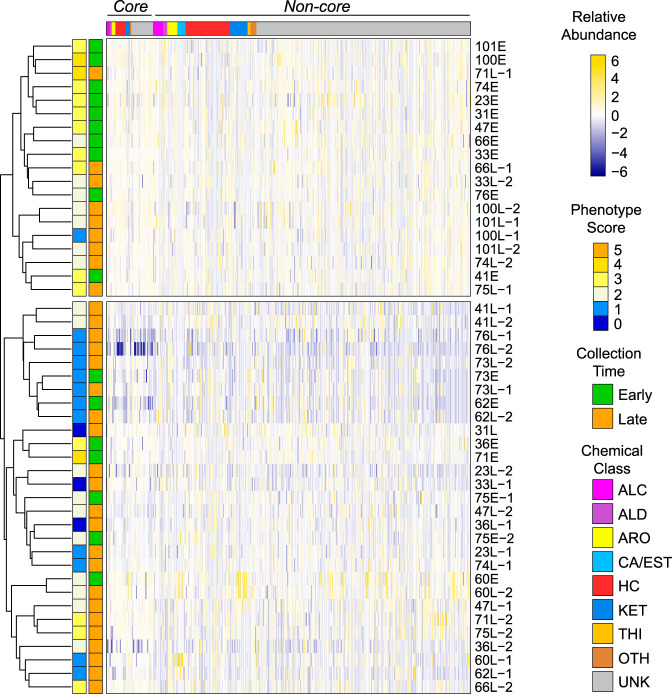
Hierarchical clustering analysis (HCA) of the truncated set of 48 P. aeruginosa clinical CF isolates, based on the relative abundance of 539 volatile compounds. Volatiles are in columns (standardized relative abundance). Clustering is based on rows (isolates), which are color coded by their phenotype score (left color block) and relative time of collection (right color block). ALC, alcohols; ALD, aldehydes; ARO, aromatics; CA/EST, carboxylic acids and esters; HC, hydrocarbons; KET, ketones; THI, thiols; OTH, other compounds; UNK, unknown identity.

**FIG 9:**
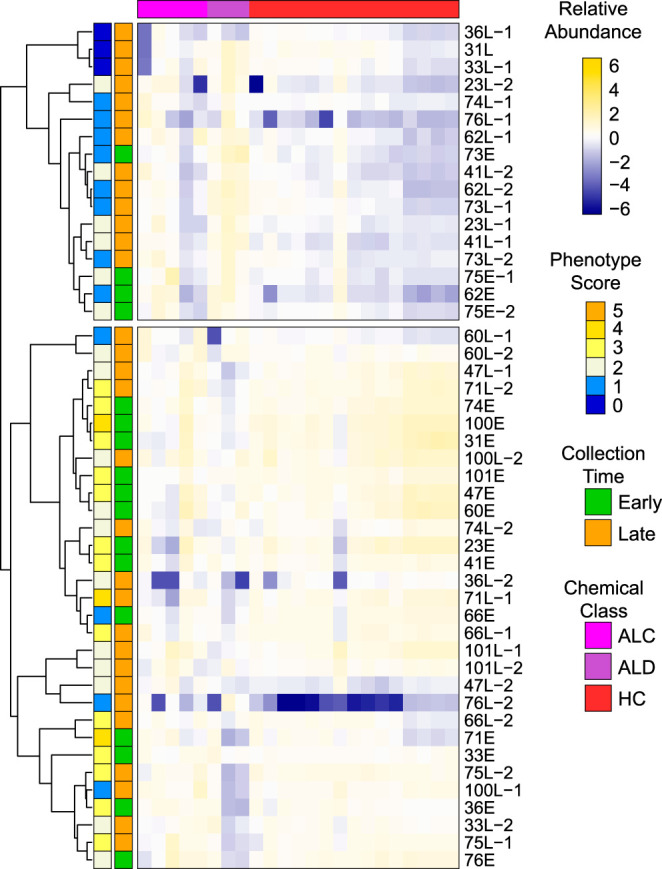
Hierarchical clustering analysis (HCA) of the truncated set of 48 P. aeruginosa clinical CF isolates, based on the relative abundance of 23 core alcohols, aldehydes, and hydrocarbons. Volatiles are in columns (standardized relative abundance). Clustering is based on rows (isolates), which are color coded by their phenotype score (left color block) and relative time of collection (right color block). ALC, alcohols; ALD, aldehydes; HC, hydrocarbons.

Though we did not observe any differences in the early- and late-isolate volatilomes based on presence and absence of metabolites, the chromatograms suggest a reduced volatilome in late infection. We therefore posited that while the numbers of volatiles do not significantly change from early to late infection isolates, the relative concentrations of volatiles do. Using volatile abundances, we performed HCA on the 20 selected early and late isolates and found clustering by the time of isolate collection and phenotype score (Fig. S4b). We were interested in whether clustering still occurs in the larger, less curated set of 48 isolates (i.e., the truncated set), which has more discordance between phenotype and time of collection (Fig. 1). HCA of the truncated set of isolates shows that early isolates are generally more similar to one another than late isolates are (Fig. 8), reinforcing observations made by calculating Euclidean distances; however, significant proportions of the early and the late isolates are misclustered. Interestingly, the majority of the late isolates clustering with the early isolates in the upper clade have more early-like phenotypes, and several early isolates that have more late-like phenotypes (e.g., 62E, 73E, and 70E) cluster in the lower clade with late isolates, indicating a relationship between the isolate phenomes and volatilomes. We also observed clustering by phenotype when the volatilomes of all 81 isolates were analyzed (Fig. S5). Similar phenomena were observed when the truncated set or all 81 isolates were clustered by the core volatilome (Fig. S6) and even when only the 23 core alcohols, aldehydes, and hydrocarbons were used (Fig. 9; Fig. S7), suggesting that a set of conserved volatiles could be identified and used as biomarkers for detecting phenotypic changes in chronic infections.

10.1128/mSphere.00843-20.5 FIG S5 Hierarchical clustering analysis (HCA) of the 81 P. aeruginosa clinical CF isolates, based on the relative abundance of 539 volatile compounds. Core compounds are present in at least 95% of isolates; noncore compounds are present in less than 95%. Volatiles are in columns (standardized relative abundance). Clustering is based on rows (isolates), which are color coded by their phenotype score (left color block) and relative time of collection (right color block). Download FIG S5, JPG file, 0.7 MB. Copyright © 2020 Davis et al. 2020 Davis et al. This content is distributed under the terms of the Creative Commons Attribution 4.0 International license.

10.1128/mSphere.00843-20.6 FIG S6 Hierarchical clustering analysis (HCA) of (a) the truncated set of 48 and (b) full set of 81 P. aeruginosa clinical CF isolates, based on the relative abundance of the 69 core volatile compounds. Volatiles are in columns (standardized relative abundance). Clustering is based on rows (isolates), which are color coded by their phenotype score (left color block) and relative time of collection (right color block). Download FIG S6, JPG file, 0.6 MB. Copyright © 2020 Davis et al. 2020 Davis et al. This content is distributed under the terms of the Creative Commons Attribution 4.0 International license.

10.1128/mSphere.00843-20.7 FIG S7 Hierarchical clustering analysis (HCA) of the 81 P. aeruginosa clinical CF isolates, based on the relative abundance of 23 core alcohols, aldehydes, and hydrocarbons. Volatiles are in columns (standardized relative abundance). Clustering is based on rows (isolates), which are color coded by their phenotype score (left color block) and relative time of collection (right color block). Download FIG S7, JPG file, 0.4 MB. Copyright © 2020 Davis et al. 2020 Davis et al. This content is distributed under the terms of the Creative Commons Attribution 4.0 International license.

## DISCUSSION

P. aeruginosa isolates from cystic fibrosis lung infections have a large and diverse volatilome. We conservatively attribute 539 detected volatiles to P. aeruginosa growth and metabolism, only 13% of which were core volatiles. For volatiles that we could assign to a chemical class, hydrocarbons represented the highest proportion, followed by ketones, which we observed both in the pan-volatilome and for individual patient isolates. Approximately 40% of core volatiles have been identified in other P. aeruginosa metabolomic studies, including the most frequently detected *in vitro* volatiles 2-butanone, 2-nonanone, dimethyl sulfide, and dimethyl disulfide (see Table 1 for references). The majority of the core volatiles we detected, however, are reported here for the first time. This opens up speculation that there may be a unique core volatile profile for strains colonizing the CF lung, though due to the interstrain variation in the P. aeruginosa volatilome, many more CF and non-CF isolates will need to be characterized via untargeted GC×GC analyses to determine if this may be the case. To date, a number of studies have attempted to identify collections of volatiles to differentiate P. aeruginosa-positive or -negative subjects from breath (44, 60–67) and bronchoalveolar lavage (25), with promising results. Unlike this study, however, the referenced analyses were targeted at single compounds (e.g., hydrogen cyanide or 2-aminoacetophenone), did not characterize the volatilome of P. aeruginosa in detail, and/or did not draw inferences to infection stage or changes across time. This study represents the first untargeted comparative analysis of P. aeruginosa volatilomes over long-term chronic infections, expanding the body of knowledge on P. aeruginosa metabolism and broadening the potential applications for breath-based diagnostics.

Despite the trend toward reduced or loss-of-phenotype expression in late isolates—which we hypothesized would correspond to a reduced metabolome size—we observed that late isolates produce the same number and variety of volatile compounds as early isolates. However, the relative abundance of volatiles produced by the late isolates is significantly reduced. Our data suggest that mutations that arise in P. aeruginosa chronic infection isolates do not result in complete inhibition of metabolic pathways but may instead reduce metabolic flux. This observation has implications for diagnostics, signifying that presence and absence of P. aeruginosa volatile metabolites would provide little diagnostic value for tracking chronic lung infections. Rather, a breath-based diagnostic for monitoring P. aeruginosa adaptations in CF will likely need to measure the relative abundance of metabolites. Importantly, a subset of core volatiles can be used to cluster isolates based on commonalities in phenotypes, indicating that a diagnostic based on the relative abundances of conserved hydrocarbons, alcohols, and aldehydes (or a few selected volatiles from these classes) may be sufficient to track P. aeruginosa infection in most patients. It should be noted that core P. aeruginosa volatiles are not necessarily unique to P. aeruginosa. In fact, a review of the mVOC 2.0 microbial volatile organic compound database (68) shows that nearly half of the core volatiles (and a third of named compounds overall) have been reported as being produced by other CF lung bacterial pathogens, including Staphylococcus aureus, Klebsiella pneumoniae, Haemophilus influenzae, Stenotrophomonas maltophilia, and *Burkholderia* spp. This further highlights the necessity of developing a breath-based diagnostic based on the relative abundances of sets of volatiles, which has been shown to accurately discriminate between human pathogens (distantly or closely related to P. aeruginosa) in numerous *in vitro*, *ex vivo*, and *in vivo* analyses (25, 48, 69–71).

Based on the genomic heterogeneity of P. aeruginosa (3, 33–35), we hypothesized that every patient would have a unique P. aeruginosa volatilome and that intrapatient isolates would have more similar volatilomes than interpatient isolates. On the contrary, we observed that the volatilomes of early and late isolates from a single patient are no more similar to one another than they are to any other isolate (intrapatient versus pooled data [Fig. 3]). Interestingly, the volatilomes of interpatient early isolates are even more similar than intrapatient isolates, while the volatilomes of interpatient late isolates have the highest dissimilarity. These data indicate that the genomic similarity within patients is less influential on the volatilome than the posttranscriptional diversity that evolves within a chronic P. aeruginosa infection population. The data from patient 23, however, suggest that intrapatient isolates are more likely to have similar volatilomes when there are fewer mutational and phenotypic differences between them. From these results, we propose that a breath-based diagnostic to track P. aeruginosa chronic lung infections would be personalized, with each patient’s breathprint at initial infection serving as a baseline to which subsequent breathprints are compared. Sustained, significant deviations from the patient’s baseline breathprint would indicate that the P. aeruginosa population phenotype has changed, and therefore, the patient may be at higher risk for associated poor clinical outcomes (e.g., higher risk of exacerbation) (15) and at higher risk of infection eradication failure after antibiotic treatment (12). In a longitudinal case study of a single CF patient, we showed that changes in the dominant species colonizing the lung are correlated with changes in the volatile metabolites detected in sputum (72), and therefore, monitoring for breathprint deviations may also work for P. aeruginosa infections, as these data suggest. Despite this, the issue of specificity becomes a concern unless biomarkers for individual P. aeruginosa phenotypes are identified and used for tracking; this is the subject of ongoing work.

We highlight some limitations in this study. Although we make reference to early isolates and those phenotypes associated with early infection, it is important to recognize that these isolates were merely the first culturable P. aeruginosa from the patients included in this study. It is likely that some patients had P. aeruginosa in the lung for some years in spite of persistent negative cultures into late childhood or adolescence (15), exemplified by the fact that isolates 62E and 73E exhibited late-like phenotypes. Phenotype discordance with collection time or apparent infection stage underscores a major drawback of culture-based methods for infection staging (e.g., *in vitro* phenotyping) in that the respiratory microbiome is prone to undersampling, especially during initial colonization, due to the complex structure of the lung. The increased use of new therapeutics (e.g., CFTR modulators) also impacts culture-based diagnostics, as they reduce sputum production, limiting the opportunity for clinical cultures. Utilizing breath-based biomarkers, on the other hand, can overcome these challenges, as breath represents a sample of the entire ventilated lung environment.

P. aeruginosa chronic infection phenotypes are positively correlated with advanced patient age (15, 73–75), and we observe a moderate but significant correlation (Kendall’s tau, –0.41; *P < *0.001) between subject age and late-infection phenotype scores in this study. The variability of isolate volatilomes we observed might therefore be explained by the age of their corresponding patients. Testing this would require analyzing isolate volatilomes from a larger number of subjects that were diagnosed with their first P. aeruginosa infections at younger and older ages. In either case, the issue of the potential covariance between age and volatilome on P. aeruginosa diagnostics could be overcome by taking a multifactorial approach as opposed to using volatile biomarkers only. We may find that using a combination of several clinical predictors, such as age, P. aeruginosa phenotype, and volatilome, provides more diagnostic power than any individual metric. It will be important to explore this more closely in future work. Additionally, while we presented volatilome trends in relation to only five clinically relevant phenotypes in this study (production of pyocyanin, rhamnolipids, and proteases, twitching mobility, and mucoidy), there are additional clinically relevant phenotypes, including antibiotic resistance, that should be included in future analyses.

In summary, current methods for tracking P. aeruginosa lung infection progression are predominantly culture based (16, 21), and our results suggest a potential role for *in vitro* volatile metabolomics in staging chronic infections in conjunction with established clinical laboratory methods. The clinically correlated P. aeruginosa phenotypes we measured in this study—mucoidy, motility, and quorum-regulated traits—are sensitive to environment (76–79); as such, the metabolomes we characterized from *in vitro* cultures are unlikely to directly reflect the metabolomes these isolates produced in the CF lung. For the development of a breath test for tracking infections *in situ*, it will be necessary to utilize culture models that are more reflective of the lung (80–83) and to collect *ex vivo* and *in vivo* infection volatilomes (25, 61, 84). The utility of breath for tracking chronic lung infections is being demonstrated in tuberculosis lung infection models (26), and therefore, we anticipate that the development of a breath-based diagnostic for tracking P. aeruginosa chronic lung infections is feasible. It will also be important to ensure that the P. aeruginosa volatiles we have identified are detectable in breath. This is the primary goal of the observational clinical study IMproving P. Aeruginosa deteCTion using Breath (IMPACT-Breath), which is under way. Despite the limitations of *in vitro* analyses, this study presents the first comprehensive analysis of the P. aeruginosa volatilome from chronic CF lung infections and lays the groundwork for the application of volatile metabolomics in tracking CF lung disease.

## MATERIALS AND METHODS

### Bacterial isolates.

Eighty one P. aeruginosa isolates from 17 individuals with CF were acquired from the Cystic Fibrosis Isolate Core at Seattle Children’s Center for Global Infectious Disease Research. From the majority of patients we obtained three isolates: one early isolate, defined as the first cultured P. aeruginosa isolate, and two late isolates collected a minimum of 5 years after the first isolate. For one patient (patient 23), 32 additional isolates were collected at intervals over 7.5 years between the collections of the early and two late isolates. For one patient, only one isolate was included in this study. The full strain names of the isolates are provided in Table S1, and all isolates are available upon request from the CF Isolate Core (https://www.seattlechildrens.org/research/resources/cystic-fibrosis-isolate/).

We quantified five phenotypes that are correlated with chronic infections: proteases, pyocyanin, rhamnolipids, twitching motility, and mucoidy. P. aeruginosa was cultured from glycerol stocks on lysogeny broth Lennox (LB) agar for 24 h, and then a single colony was cultured aerobically to stationary phase in LB broth (10 g tryptone, 5 g yeast extract, 5 g NaCl per liter) at 37°C with shaking at 200 rpm, unless otherwise noted. Results of phenotype assays are reported in Table S1 as means for biological triplicates, except for mucoidy, for which five replicates were measured. Pyocyanin production was evaluated by methods adapted from reference 85. Cell-free P. aeruginosa culture supernatant (7.5 ml) was extracted with 4.5 ml of chloroform, inverted for 2 min and centrifuged at 4,122 × *g* for 15 min. Three milliliters of the organic phase was removed, extracted with 750 μl of 0.2 *N* hydrochloric acid, and vortexed for 2 min. The aqueous phase was aliquoted into a 96-well plate, and absorbance was measured at 520 nm. Protease production was evaluated by methods adapted from reference 15. A sterile wooden inoculation stick was dipped into a culture of P. aeruginosa and then gently touched to the surface of a brain heart infusion-skim milk agar (1.5%) plate. Plates were incubated upright at 37°C, and zones of clearance were measured at 48 h. Rhamnolipid production was evaluated by methods adapted from reference 86. Proteose peptone-glucose-ammonium salts (PPGAS) medium was inoculated from an LB preculture of P. aeruginosa and cultured at 37°C to stationary phase with shaking at 200 rpm. Cultures were centrifuged at 21,694 × *g* for 1 min to pellet cells. The supernatant was serially diluted 2-fold in PPGAS, and 20 μl of each dilution was spotted onto a microtiter plate lid. Spots were classified as drops or collapsed drops and assigned numeric scores corresponding to the number of dilutions needed to obtain a drop. Twitching motility was evaluated by methods adapted from references 87 and 88. The pointed end of a sterile toothpick was touched to the edge of a single P. aeruginosa colony and then stabbed to the bottom of a twitching motility agar plate (per liter: 10 g tryptone, 5 g NaCl, 5 g yeast extract, 10 g agar). Plates were incubated at 37°C for 24 h, and the radius of the interstitial biofilm was measured. For mucoidy, frozen glycerol stocks of P. aeruginosa were streaked onto LB agar (1.5%) plates and grown for 48 h. Colonies were visually inspected, and the degree of mucoid morphology was scored from 0 to 2, in 0.5 increments (0 = highly mucoid, 2 = nonmucoid). Twenty pairs of isolates (one early and one late) from 10 patients were selected for additional statistical analyses, described below, based on changes to their phenotypes during chronic infections.

### Sample preparation.

Isolates were cultured as previously described (89). Briefly, isolates were cultured aerobically for 16 h at 37°C in 5 ml of LB and then diluted 1,000-fold into 25 ml of fresh LB and grown for 24 h under the same conditions. For metabolomics analyses, cells were removed via centrifugation through a 0.2 μm filter, and 2 ml of each filtrate was transferred to a 10 ml GC headspace vial with a screw cap. Samples were prepared in biological triplicate, with LB medium controls prepared in parallel, and stored at −20°C prior to analysis.

### GC×GC analysis and data processing.

Culture filtrates were thawed and maintained at 4°C until analyzed, as previously described (89). Headspace volatiles were characterized using a Pegasus 4D GC×GC–TOF-MS (LECO Corporation, St. Joseph, MI) equipped with a MPS Pro rail autosampler (Gerstel Inc., Linthicum Heights, MD). Column set configuration and GC×GC, MS, and data processing method parameters were previously reported (89) and are summarized in Table 1 of Miscellaneous Information (https://doi.org/10.6084/m9.figshare.12990908).

Data processing steps are outlined in Fig. 1 of Miscellaneous Information. Chromatographic artifacts and suspected contaminants based on peak names and comparisons to blanks were removed (see Table 2 in Miscellaneous Information), as well as poorly modulated peaks eluting prior to 358 s (acetone retention time) in the first dimension. Missing values were handled as follows: if detected in one of three replicates, the measured value was permuted to 0; if detected in two of three replicates, the missing value was imputed as half of the minimum detected value for that compound across all samples.

Probabilistic quotient normalization (PQN) was applied to account for differences in peak abundance due to variations in culture cell density, followed by a log_10_ transformation (90). Peaks were further filtered out based on the following criteria: (i) a within-triplicate intraclass correlation coefficient (ICC) of <0.75 (definition, absolute agreement; model, two-way mixed effects; type, mean of *k* measurements) (91) and (ii) detection only in sterile medium or abundance not significantly greater than in sterile medium using the Wilcoxon rank-sum test with Benjamini-Hochberg adjustment (significance threshold of 0.05) (92).

For statistical analyses beyond the reporting of detected peaks, peaks that showed significant correlations to run order were identified using Kendall’s tau with Benjamini-Hochberg adjustment (−0.6 ≤ τ ≥ 0.6, significance threshold of 0.05) and removed. Principal component analysis (PCA) revealed an apparent batch effect that was attributed to, at least in part, a nonbiological phenomenon and was described previously (89). This batch effect was corrected using an empirical Bayes approach (93). The geometric means of triplicates of the batch-corrected data were used for further statistical analyses. For analysis of the 10 paired early and late chronic infection isolates, batch-corrected data were not used, as all of these isolates were contained within the same batch.

Peaks were putatively identified using published reporting standards (94). Level 2 identifications were determined based on the following criteria: (i) ≥80% mass spectral forward match using the NIST 2011 library; (ii) (a) first-dimension retention times that possess a strong linear fit with carbon number in homologous series (*r*^2^ ≥ 0.995, for the identification of selected 2-, 3-, and 4-ketones) or (b) experimentally determined linear retention indices (LRIs) consistent with published LRIs, as determined by the following acceptance criteria based on the differences between experimental and published nonpolar and polar column RIs for Grob’s test mix: [(RI_experimental_ − RI_nonpolar_)/(RI_polar_ − RI_nonpolar_)] × 100 = 0 to 43%.

Level 3 identifications were based on at least an 80% mass-spectral forward match score. Chemical classifications (alcohols, aldehydes, aromatics, carboxylic acids, esters, hydrocarbons, ketones, thiols, or other) were assigned based primarily on their mass spectral identity and secondarily on their chromatographic characteristics. Using polar second-dimension columns, compounds of different functional classes elute at easily discernible second-dimension retention times (^2^*t*_R_) in a stratified manner, typically as follows (from smallest ^2^*t*_R_ to largest): hydrocarbons < ketones < aromatics (43, 95–98). In this study, compounds identified as hydrocarbon, ketones, and aromatics eluted at approximately 0.71 s ± 0.05 s, 0.88 s ± 0.22 s, and 1.12 s ± 0.26 s, respectively. Level 4 identifications are those that meet none of the above-mentioned criteria.

### Statistical analyses.

A phenotype score was determined, for each replicate, by scaling the data for each phenotype to a range of 0 to 1, where 1 is the maximum value and 0 is the minimum value (excluding outliers). Scaled phenotype data were then summed to yield the score and averaged across replicates. Patient age was not included in calculation of scores. Two-tailed Wilcoxon signed-rank tests (with continuity correction, where appropriate; significance threshold of 0.05) were used to test for significant differences between late- and early-isolate scores.

Volatile compounds that were detected in at least 95% of all samples were classified as “core” volatiles. Significant differences between interisolate phenotypes were tested using the Wilcoxon signed-rank test (significance threshold of 0.05). Significant differences between intra- and interpatient volatilomes were tested using one-way analysis of variance (ANOVA) (significance threshold of 0.05), and Tukey’s honestly significant differences (HSD) multiple comparisons procedure (significance threshold of 0.05). The relatedness of isolates based on their volatilomes was assessed using agglomerative hierarchical clustering analysis (HCA), nonmetric multidimensional scaling (NMDS), and permutational multivariate analysis of variances (PERMANOVA) on the Euclidean distances between isolates. Linear regression and Pearson’s correlation of Euclidean distances of patient 23 isolates were used to assess intrapatient isolate dissimilarity over time. All statistical analyses were performed using R version 3.3.2 (The R Foundation for Statistical Computing) with the following packages (version): ICC (2.3.0), pairwiseAdonis (0.0.1), pheatmap (1.0.8), stats (3.3.2), and sva (3.22.0).

### Data availability.

Metabolomic data (chemical feature peak areas and retention time information) included in this study are available at the NIH Common Fund’s National Metabolomics Data Repository (NMDR) website, the Metabolomics Workbench, at www.metabolomicsworkbench.org, where it has been assigned project ID PR000970 and study ID ST001414 (https://doi.org/10.21228/M89Q4F). Miscellaneous Information (additional tables and figures) can be found at Figshare (https://doi.org/10.6084/m9.figshare.12990908).
