# Supplementary material for: Pseudomonas aeruginosa Volatilome Characteristics and Adaptations in Chronic Cystic Fibrosis Lung Infections
Source: mSphere. 2020 Oct 7;5(5):e00843-20. doi: 10.1128/mSphere.00843-20 (PMC7568651; doi:10.1128/mSphere.00843-20)
Supplement: TABLE S1 [file mSphere.00843-20-st001.pdf]

Table S1.

Isolate	Isolate Core Code[a]	E/L/I	AGE	PYO	PRO	RHL	TWI	MUC	Score	Significance
**21**		N/A	N/A	0.049	0.00	1	0.05	0.5	1	
**23E***	AMT0023-30	E	0.5	0.609	1.08	5	0.43	0.92	3	
**23I-1**	AMT0023-10	I	0.75	0.661	1.23	4	0.54	0.83	4	*P* = 1
**23I-2**	AMT0023-33	I	1	0.592	0.77	4	0.07	1.00	3	*P* ≈ .37
**23I-3**	AMT0023-25	I	1	0.576	0.83	3	0.10	1.00	2	*P* ≈ .35
**23I-4**	AMT0023-26	I	1.25	0.793	0.85	3	0.00	1.00	3	*P* ≈ .15
**23I-5**	AMT0023-12	I	1.5	0.754	1.17	3	0.00	1.00	3	*P* = .59
**23I-6**	AMT0023-20	I	1.75	0.867	0.43	3	0.03	1.00	2	*P* = .15
**23I-7**	AMT0023-18	I	1.75	0.566	0.72	4	0.10	1.00	3	*P* ≈ .15
**23I-8**	AMT0023-2	I	2	0.700	1.10	4	0.00	1.00	3	*P* ≈ 1
**23I-9**	AMT0023-27	I	2	0.491	0.53	3	0.00	1.00	2	*P* ≈ .17
**23I-10**	AMT0023-14	I	2	0.531	1.35	3	0.00	0.92	4	*P* ≈ .37
**23I-11**	AMT0023-21	I	2	0.530	0.87	2	0.03	1.00	3	*P* ≈ .17
**23I-12**	AMT023-11	I	2	0.565	0.37	3	0.00	1.00	2	*P* = .17
**23I-13**	AMT0023-22	I	2	0.622	0.60	3	0.00	1.00	2	*P* ≈ .15
**23I-14**	AMT0023-23	I	2.25	0.457	0.00	3	0.00	1.00	2	*P* ≈ .17
**23I-15**	AMT0023-19	I	2.25	0.862	1.20	4	0.00	1.00	3	*P* ≈ .35
**23I-16**	AMT0023-6	I	2.25	0.540	1.10	4	0.22	0.83	3	*P* ≈ .77
**23I-17**	AMT0023-24	I	2.5	0.586	0.87	2	0.48	0.50	3	*P* ≈ .15
**23I-18**	AMT0023-15	I	2.5	0.882	1.67	2	0.22	0.92	4	*P* ≈ .35
**23I-19**	AMT0023-9	I	2.5	0.397	0.95	3	0.00	1.00	2	*P* ≈ .37
**23I-20**	AMT0023-16	I	2.5	0.998	1.10	2	0.00	1.00	3	*P* ≈ .35
**23I-21**	AMT0023-13	I	2.75	0.837	1.13	3	0.00	1.00	3	*P* ≈ .35
**23I-22**	AMT0023-17	I	2.75	1.061	1.10	3	0.05	1.00	3	*P* ≈ .15
**23I-23**	AMT0023-7	I	2.75	0.596	1.20	4	0.41	0.92	3	*P* ≈ 1
**23I-24**	AMT0023-3	I	3	0.774	1.08	3	0.00	1.00	3	*P* ≈ 1
**23I-25**	AMT0023-8	I	3	0.676	1.30	3	0.39	0.92	3	*P* ≈ 1
**23I-26**	AMT0023-4	I	3	0.530	1.02	3	0.48	1.00	3	*P* = 1
**23I-27**	AMT0023-5	I	3	0.527	1.02	3	0.03	1.00	3	*P* = .35
**23I-28**	AMT0023-1	I	3	0.538	0.93	3	0.00	1.00	3	*P* ≈ .37
**23I-29**	AMT0023-28	I	3	0.785	0.27	4	0.17	1.00	2	*P* = .37
**23I-30**	AMT0023-32	I	5	0.711	0.80	2	0.05	0.83	2	*P* = .37
**23I-31**	AMT0023-31	I	7.6	0.030	0.30	1	0.00	1.00	1	*P* ≈ .25
**23I-32**	AMT0023-29	I	7.6	0.066	0.32	1	0.00	0.92	1	*P* ≈ .25
**23L-1***	AMT0023-34	L	8	0.042	0.22	2	0.05	0.92	1	*P* ≈ .17
**23L-2**	AMT0023-35	L	8	0.174	0.30	2	0.00	1.00	2	*P* = .25
**31E***	AMT0031-2	E	6.3	0.840	0.93	5	0.21	0.75	3	
**31L-1***	AMT0031-1	L	12.8	0.106	0.00	0	0.17	0.00	0	*P* ≈ .25

**33E***	AMT0033-2	E	1.1	0.614	0.77	5	0.38	0.75	3	
**33L-1**	AMT0033-1	L	13.2	0.003	0.00	0	0.00	0.17	0	*P* = .25
**33L-2***	AMT0033-3	L	13.2	0.145	0.23	1	0.20	1.00	2	*P* = .37
**36E***	AMT0036-3	E	6.7	0.902	0.65	5	0.00	1.00	3	
**36L-1***	AMT0036-1	L	15.4	0.531	0.00	0	0.00	0.25	0	*P* = .17
**36L-2**	AMT0036-2	L	15.4	1.492	0.00	2	0.00	1.00	2	*P* = .15
**41E***	AMT0041-1	E	5.6	0.636	0.87	4	0.03	1.00	3	
**41L-1**	AMT0041-2	L	12.8	1.112	0.00	4	0.03	1.00	2	*P* ≈ 1
**41L-2***	AMT0041-3	L	12.8	0.103	0.00	3	0.03	1.00	2	*P* ≈ .37
**47E**	AMT0047-2	E	0.8	0.504	0.57	5	0.23	1.00	3	
**47L-1**	AMT0047-1	L	6.8	0.725	0.32	5	0.03	0.17	2	*P* ≈ .17
**47L-2**	AMT0047-3	L	7.3	0.212	0.00	5	0.25	0.75	2	*P* ≈ .17
**60E**	AMT0060-3	E	7.7	0.541	0.80	1	0.20	1.00	2	
**60L-1**	AMT0060-2	L	15.4	0.267	0.00	1	0.10	0.33	1	*P* = .17
**60L-2**	AMT0060-1	L	15.4	0.342	0.45	2	0.23	1.00	2	*P* = 1
**62E**	AMT0062-2	E	4.4	0.001	0.00	0	0.00	0.92	1	
**62L-1**	AMT0062-3	L	13.1	0.096	0.00	0	0.00	0.92	1	N/A
**62L-2**	AMT0062-1	L	13.1	0.083	0.00	1	0.00	0.92	1	N/A
**66E***	AMT0066-3	E	7.2	0.569	0.55	2	0.20	1.00	2	
**66L-1***	AMT0066-2	L	13.7	1.233	0.42	3	0.23	0.83	3	*P* ≈ 1
**66L-2**	AMT0066-1	L	15.2	1.469	0.47	3	0.28	0.67	3	*P* ≈ 1
**71E**	AMT0071-2	E	3	1.827	1.70	4	0.30	0.92	4	
**71L-1**	AMT0071-1	L	9.1	3.177	1.47	3	0.18	1.00	4	*P* = 1
**71L-2**	AMT0071-3	L	9.1	1.611	1.62	3	0.37	0.75	3	*P* ≈ 1
**73E**	AMT0073-3	E	8.6	0.083	0.22	2	0.00	1.00	1	
**73L-1**	AMT0073-2	L	21.1	0.418	0.00	1	0.03	1.00	1	N/A
**73L-2**	AMT0073-1	L	21.1	0.041	0.00	1	0.03	1.00	1	*P* ≈ .35
**74E**	AMT0074-1	E	9.2	0.656	1.20	2	0.38	0.67	3	
**74L-1**	AMT0074-2	L	19.6	0.500	0.00	1	0.03	0.50	1	p ≈ .15
**74L-2**	AMT0074-3	L	19.6	0.677	0.47	2	0.05	1.00	2	p ≈ .35
**75E-1***	AMT0075-3	E	7	0.249	0.35	4	0.18	1.00	2	
**75E-2**	AMT0075-1	E	7.1	0.053	0.20	4	0.08	1.00	2	
**75L-1***	AMT0075-2	L	17.6	0.830	0.58	4	0.27	0.83	3	*P* = 1;<br>*P* = 1
**75L-2**	AMT0075-4	L	23.4	0.721	0.38	4	0.28	1.00	3	*P* = 1;<br>*P* = 1

**76E***	AMT0076-3	E	10.8	0.898	0.98	5	0.10	0.50	2	
**76L-1**	AMT0076-1	L	19.6	0.108	0.00	0	0.00	1.00	1	*P* = .35
**76L-2***	AMT0076-2	L	19.6	0.075	0.13	1	0.00	1.00	1	*P* ≈ .37
**100E***	NC-AMT0100-2	E	2.3	0.769	0.68	5	0.68	1.00	4	
**100L-1***	NC-AMT0100-1	L	9.6	0.870	0.42	1	0.00	0.33	1	*P* = .25
**100L-2**	NC-AMT0100-3	L	9.6	0.433	0.50	5	0.10	0.92	2	*P* ≈ .17
**101E***	NC-AMT0101-3	E	1	0.594	0.85	5	0.53	0.83	3	
**101L-1**	NC-AMT0101-2	L	9.6	0.673	0.60	4	0.03	1.00	2	*P* ≈ .78
**101L-2***	NC-AMT0101-3	L	9.6	0.538	0.03	3	0.00	1.00	2	*P* ≈ .37

[a] Isolate Core Code refers to the strain name assigned by the Cystic Fibrosis Isolate Core at Seattle Children's Center for Global Infectious Disease Research. Isolates are available to researchers by request to the CF Isolate Core: https://www.seattlechildrens.org/research/resources/cystic-fibrosis-isolate/
