# Supplementary figures and images for: Pseudomonas aeruginosa Volatilome Characteristics and Adaptations in Chronic Cystic Fibrosis Lung Infections
Source: mSphere. 2020 Oct 7;5(5):e00843-20. doi: 10.1128/mSphere.00843-20 (PMC7568651; doi:10.1128/mSphere.00843-20)

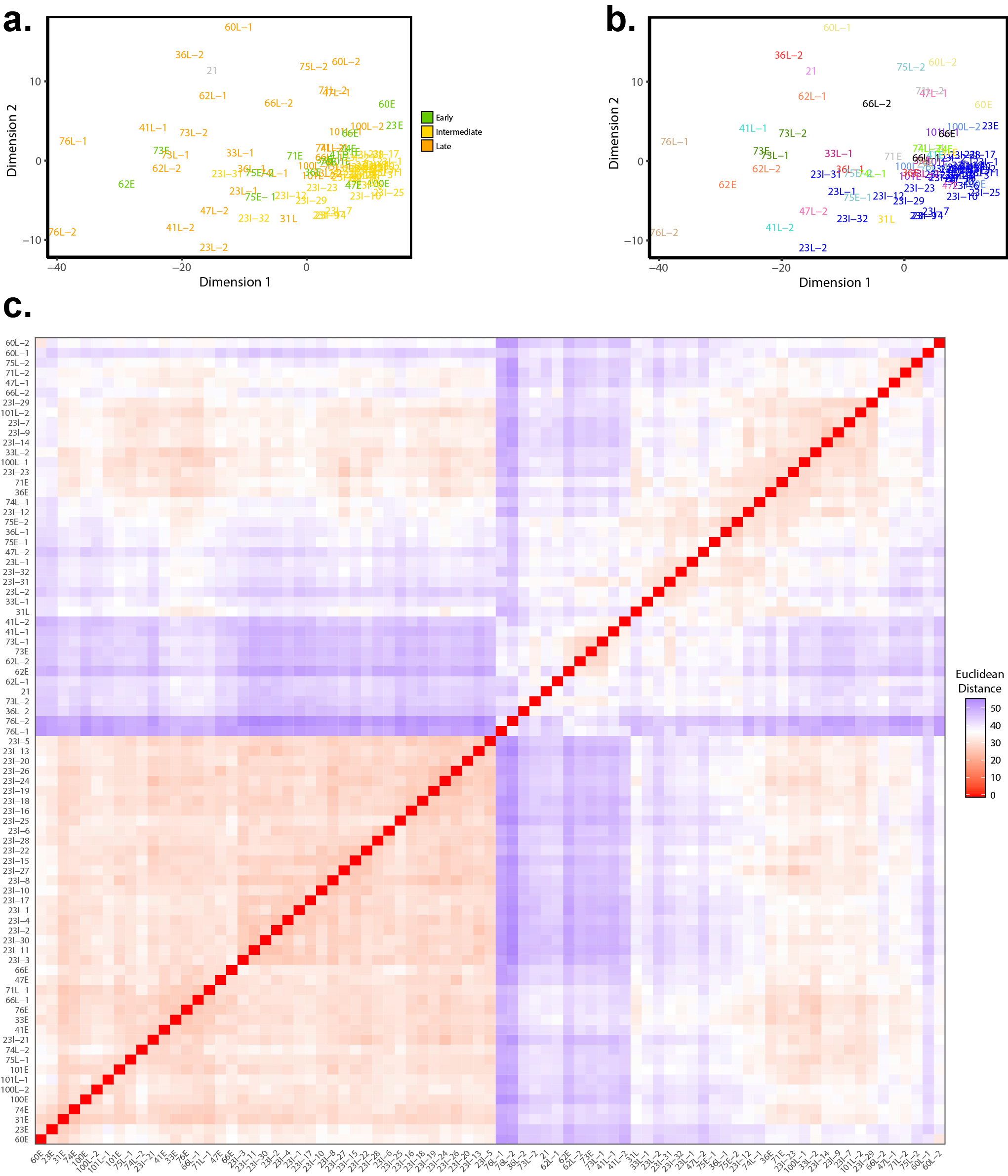

Supplement: FIG S1 [file mSphere.00843-20-sf001.jpg]

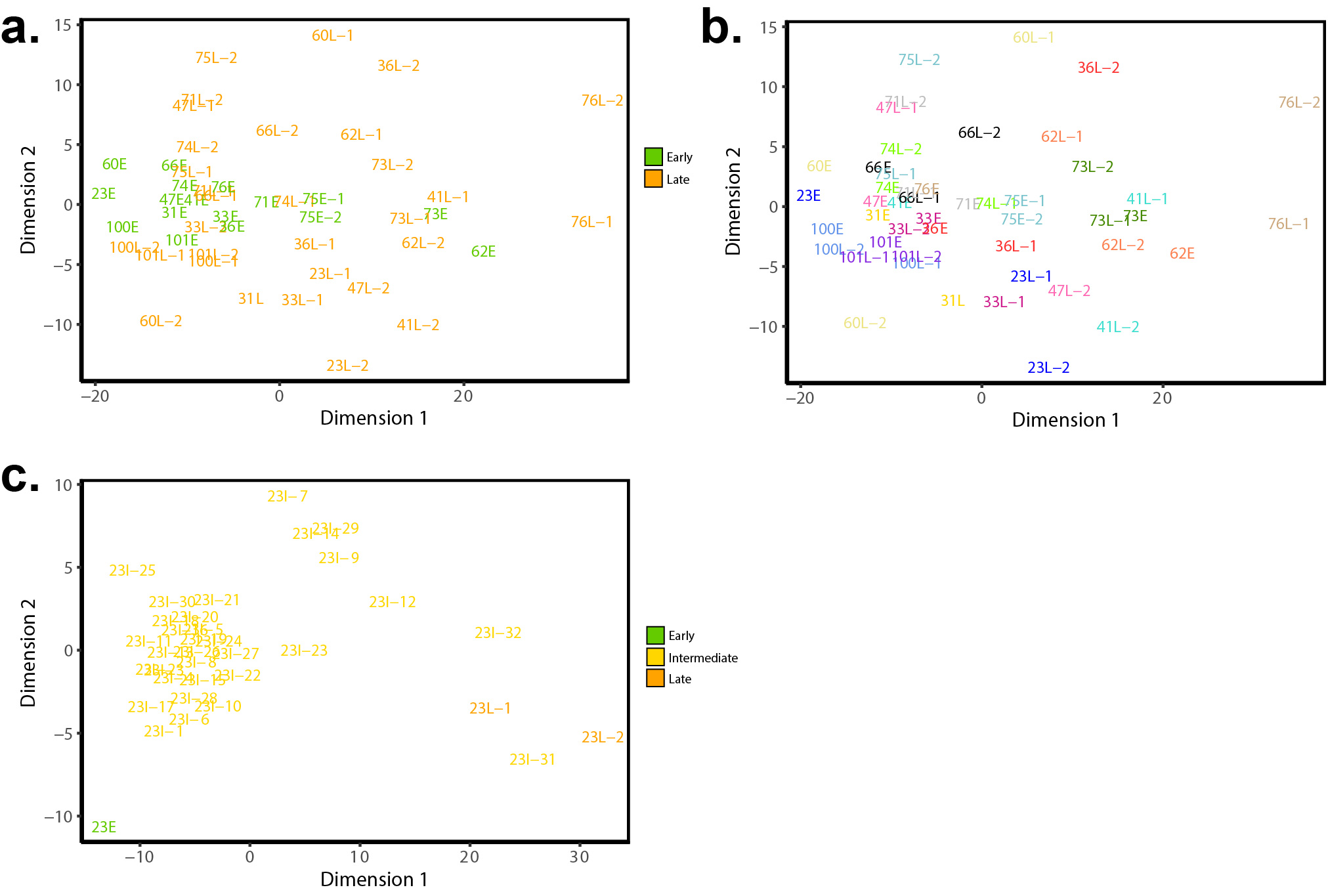

Supplement: FIG S2 [file mSphere.00843-20-sf002.jpg]

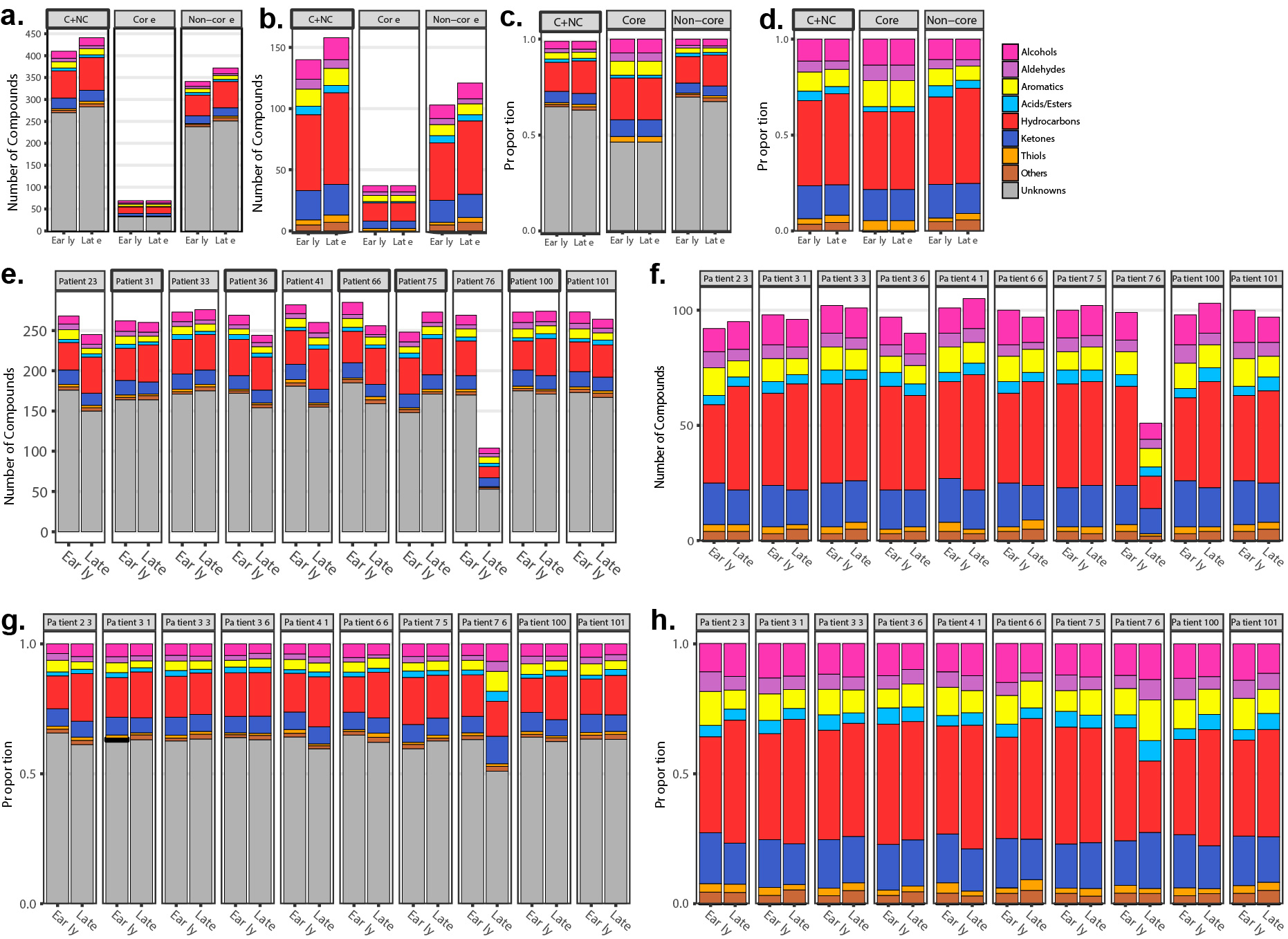

Supplement: FIG S3 [file mSphere.00843-20-sf003.jpg]

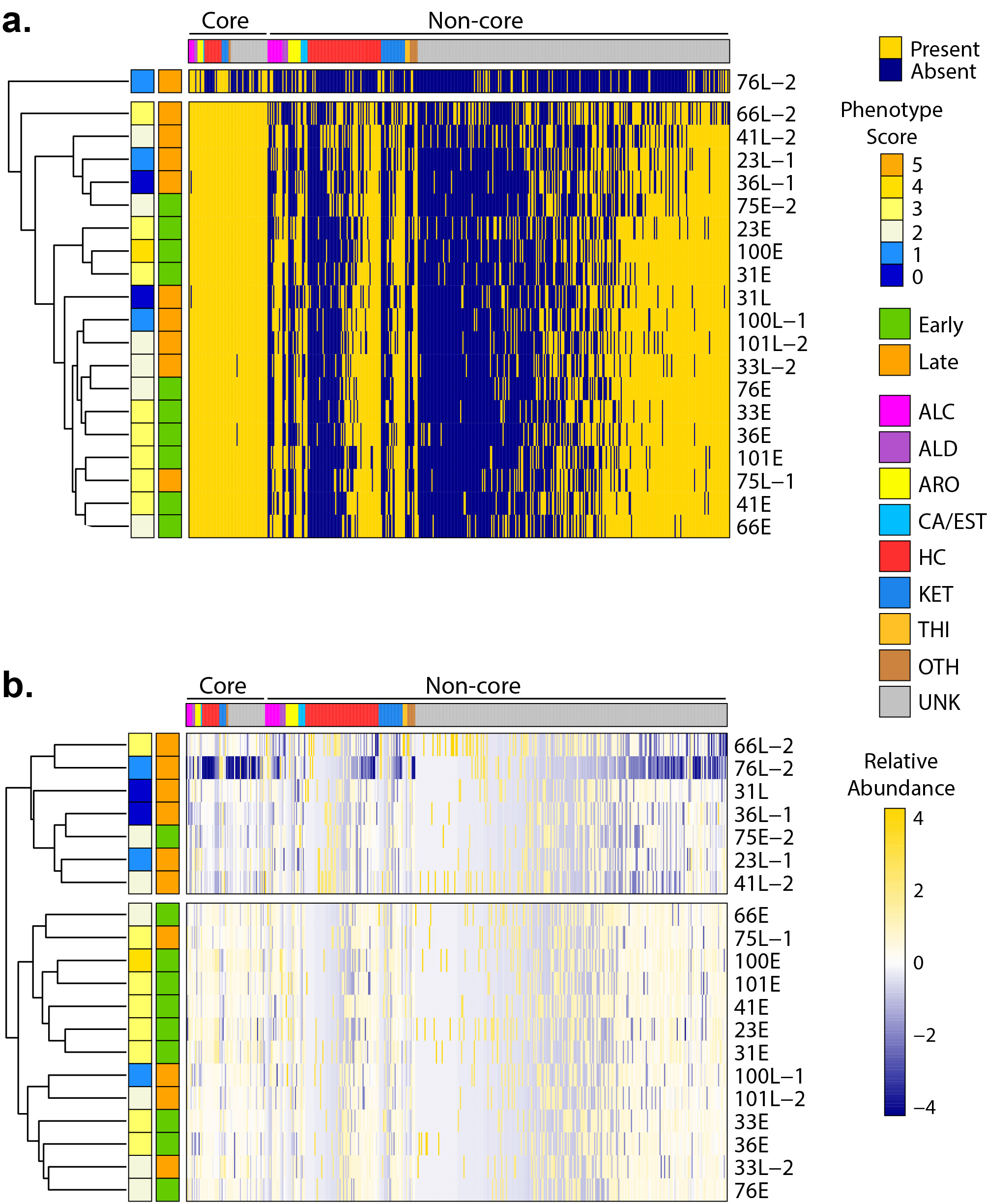

Supplement: FIG S4 [file mSphere.00843-20-sf004.jpg]

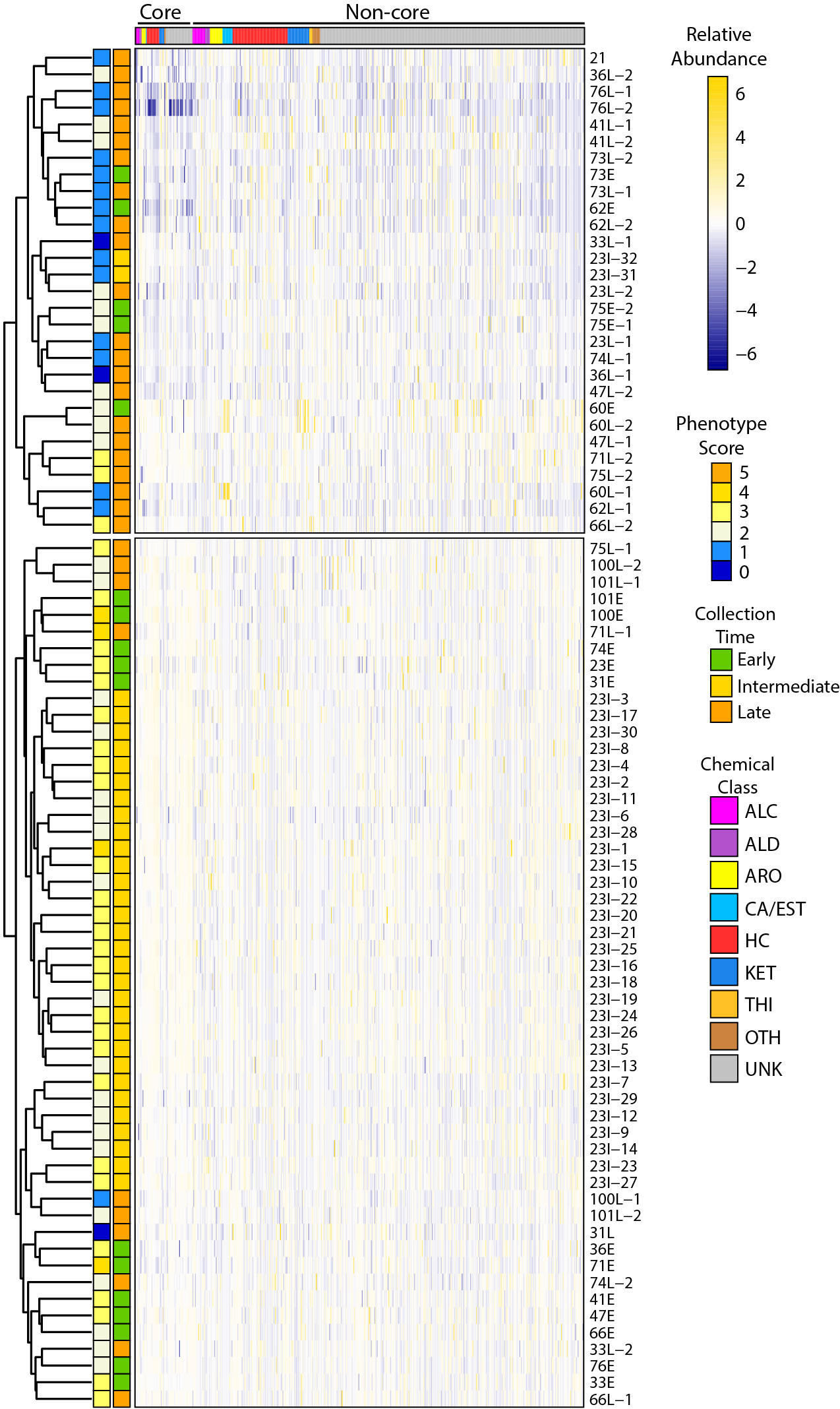

Supplement: FIG S5 [file mSphere.00843-20-sf005.jpg]

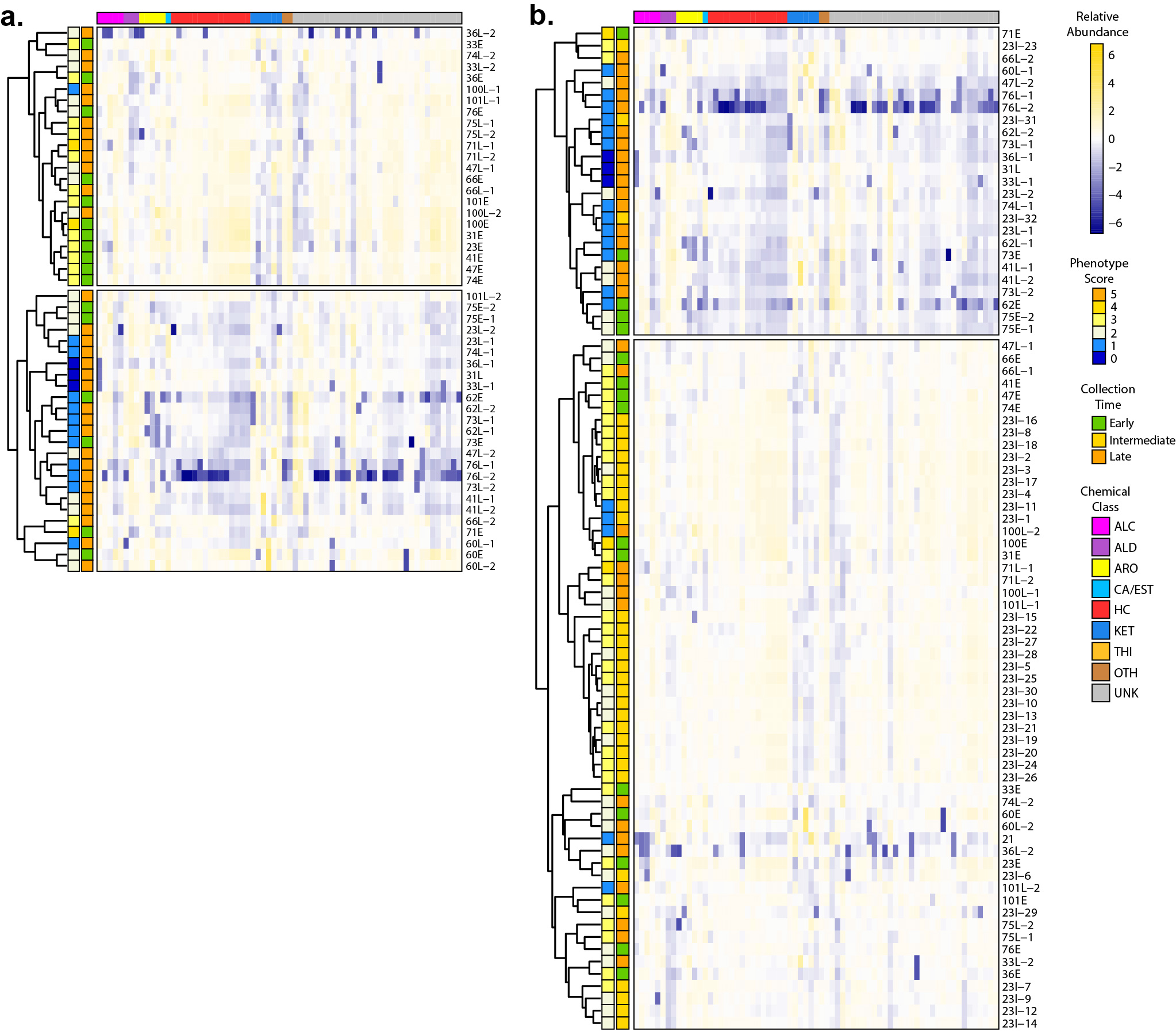

Supplement: FIG S6 [file mSphere.00843-20-sf006.jpg]

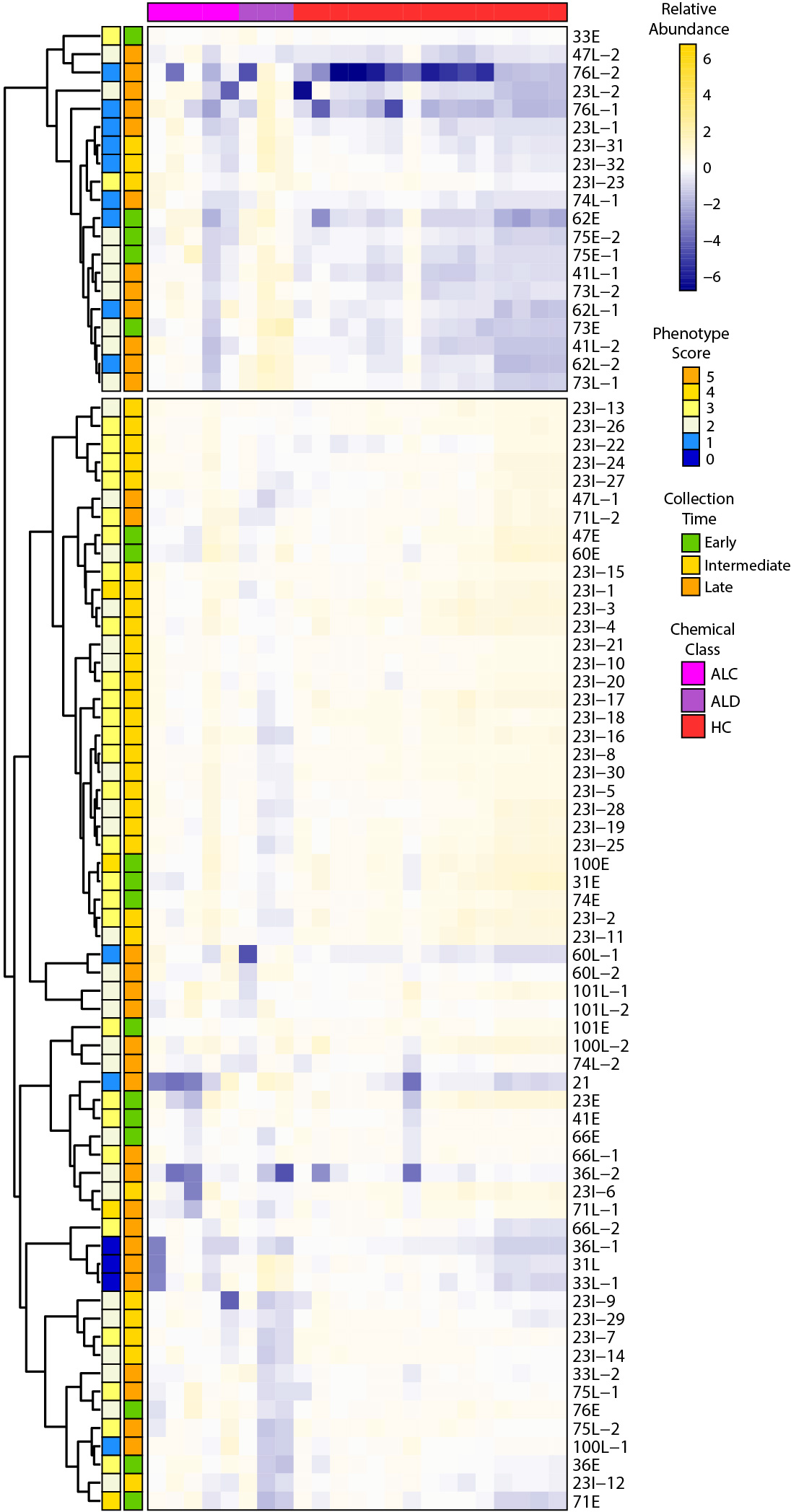

Supplement: FIG S7 [file mSphere.00843-20-sf007.jpg]
