# Supplementary material for: Pseudomonas aeruginosa Volatilome Characteristics and Adaptations in Chronic Cystic Fibrosis Lung Infections
Source: mSphere. 2020 Oct 7;5(5):e00843-20. doi: 10.1128/mSphere.00843-20 (PMC7568651; doi:10.1128/mSphere.00843-20)
Supplement: TABLE S3 [file mSphere.00843-20-st003.pdf]

Table S3.

Isolate		C + NC		Core (C)		Non-core (NC)	
		Richness	*Diversity*	*Richness*	*Diversity*	*Richness*	*Diversity*
**Pooled**	*Early*	9	1.68	7	1.66	9	1.65
	Late	9	1.64	7	1.66	9	1.60
**P23**	*Early*	9	1.77	7	1.66	9	1.79
	Late	8	1.63	7	1.66	8	1.53
**P31**	*Early*	9	1.71	7	1.66	9	1.69
	Late	8	1.61	7	1.66	7	1.45
**P33**	*Early*	9	1.70	7	1.66	9	1.67
	Late	9	1.68	7	1.66	9	1.61
**P36**	*Early*	9	1.62	7	1.66	8	1.49
	Late	9	1.68	7	1.66	8	1.58
**P41**	*Early*	9	1.71	7	1.66	9	1.69
	Late	9	1.63	7	1.66	8	1.53
**P66**	*Early*	9	1.72	7	1.66	8	1.67
	Late	9	1.65	7	1.66	7	1.54
**P75**	*Early*	9	1.68	7	1.66	8	1.59
	Late	8	1.68	7	1.66	9	1.64
**P76**	*Early*	9	1.69	7	1.66	9	1.64
	Late	9	1.90	7	1.78	8	1.87
**P100**	*Early*	9	1.78	7	1.66	9	1.80
	Late	9	1.69	7	1.66	8	1.61
**P101**	*Early*	9	1.78	7	1.66	9	1.81
	Late	9	1.74	7	1.66	9	1.72
